# Neuroimmune modulating and energy supporting nanozyme-mimic scaffold synergistically promotes axon regeneration after spinal cord injury

**DOI:** 10.1186/s12951-024-02594-2

**Published:** 2024-07-05

**Authors:** Genjiang Zheng, Wei Yu, Zeng Xu, Chen Yang, Yunhao Wang, Zhihao Yue, Qiangqiang Xiao, Wenyu Zhang, Xiaodong Wu, Fazhi Zang, Jianxi Wang, Lei Wang, Wei-En Yuan, Bo Hu, Huajiang Chen

**Affiliations:** 1 Spine Center, Department of Orthopedics, Changzheng Hospital, Naval Medical University, No. 415 Fengyang Road, Shanghai, 200003 China; 2 Shanghai Frontiers Science Center of Drug Target Identification and Delivery, School of Pharmaceutical Sciences, Shanghai Jiao Tong University, No. 800 Dongchuan Road, Shanghai, 200240 China; 3 National Key Laboratory of Innovative Immunotherapy, Shanghai Jiao Tong University, No. 800 Dongchuan Road, Shanghai, 200240 China; 4 Engineering Research Center of Cell & Therapeutic Antibody, School of Pharmacy, Ministry of Education, Shanghai Jiao Tong University, No. 800 Dongchuan Road, Shanghai, 200240 China; 5 Inner Mongolia Research Institute of Shanghai Jiao Tong University, Shanghai, 200240 China; 6 Division of Orthopaedics and Traumatology, Department of Orthopedics, Nanfang Hospital, Southern Medical University, No. Guangzhou North Road, Guangzhou, 510515 China

**Keywords:** Cerium oxide nanoparticles, Calcitonin gene-related peptide, Polarization of macrophages, Mitochondria, Reactive oxygen species, Axonal regeneration

## Abstract

Spinal cord injury (SCI) represents a profound central nervous system affliction, resulting in irreversibly compromised daily activities and disabilities. SCI involves excessive inflammatory responses, which are characterized by the existence of high levels of proinflammatory M1 macrophages, and neuronal mitochondrial energy deficit, exacerbating secondary damage and impeding axon regeneration. This study delves into the mechanistic intricacies of SCI, offering insights from the perspectives of neuroimmune regulation and mitochondrial function, leading to a pro-fibrotic macrophage phenotype and energy-supplying deficit. To address these challenges, we developed a smart scaffold incorporating enzyme mimicry nanoparticle-ceriumoxide (COPs) into nanofibers (NS@COP), which aims to pioneer a targeted neuroimmune repair strategy, rescuing CGRP receptor on macrophage and concurrently remodeling mitochondrial function. Our findings indicate that the integrated COPs restore the responsiveness of pro-inflammatory macrophages to calcitonin gene-related peptide (CGRP) signal by up-regulating receptor activity modifying protein 1 (RAMP1), a vital component of the CGRP receptor. This promotes macrophage fate commitment to an anti-inflammatory pro-resolution M2 phenotype, then alleviating glial scar formation. In addition, NS@COP implantation also protected neuronal mitochondrial function. Collectively, our results suggest that the strategy of integrating nanozyme COP nanoparticles into a nanofiber scaffold provides a promising therapeutic candidate for spinal cord trauma via rational regulation of neuroimmune communication and mitochondrial function.

**Supplementary Information:**

The online version contains supplementary material available at 10.1186/s12951-024-02594-2.

## Background

The spinal cord is an integral part of the central nervous system, playing a vital role in transmitting information between the brain and effector tissues, as well as coordinating reflexes. Unfortunately, traumatic spinal cord injury (SCI) remains a prevalent and debilitating condition, with limited treatment options available. Each year, approximately 27 million individuals worldwide suffer from long-term paralysis and mental disorders following SCI [1]. Most cases of SCI are anatomically incomplete, meaning that some connections in the neuronal routes adjacent to the lesion site are spared. However, current clinical therapies often fail to provide significant functional recovery. This is largely due to the formation of excessive glial scar tissue and impaired axon growth [2, 3].

After SCI, the harsh microenvironment triggers the accumulation and activation of pro-inflammatory macrophages (M1 macrophages), which contribute to the formation of glial scar tissue. Reactive oxygen species (ROS) play a key role in this process by promoting the accumulation of pro-inflammatory macrophages rather than pro-resolution (M2 macrophage) and driving their fate towards a pro-fibrotic phenotype, hindering functional recovery [4–8]. However, ROS does not have a direct ligand-to-receptor mechanism to enable their direct function to affect macrophage fate [9–12].

Recent studies have uncovered that sensory nerve-immune communications are the key microenvironmental cues in driving macrophage fate toward a pro-resolution phenotype [13–15], which may orchestrate axon repair after SCI through the release of protective cytokines such as IL-4 and IL-10. Previous studies showed that CGRP positive neuronal fibers undergo sprouting following SCI [16, 17]. The RAMP1, a vital component of the CGRP receptor, is abundantly expressed in macrophages, which makes the macrophages can effectively sense neuronal regulation under physiological conditions [15, 18]. However, macrophages lose their response to neuromodulation in the oxidative stress environment, as evidenced by a shift in the majority of macrophages toward a pro-inflammatory phenotype [19]. Therefore, we hypothesized that excessive ROS hijacks the CGRP/RAMP1 axis-dependent cross-talk between sensory nerves and macrophages, leading to a switch in macrophage fate towards pro-fibrotic commitment and facilitating glial scar formation.

In recent years, inorganic nanomaterials with a variety of enzyme-mimic activities, called nanozymes, have attracted significant attention in biomedicine. Compared with natural enzymes, nanozymes have the advantages of sustained activity, controllable release, multifunctional activity [20]. We designed a facile strategy to incorporate cerium oxide nanoparticles (COPs), acting as classical antioxidant nanozymes, into polycaprolactone (PCL) porous nano-scaffolds using the electrospinning technique to instantly, directly, and sustainedly cleave ROS, due to automatic shifting between Ce^4+^ and Ce^3+^ oxidation states in COPs [19, 21–24].

**Scheme 1:**
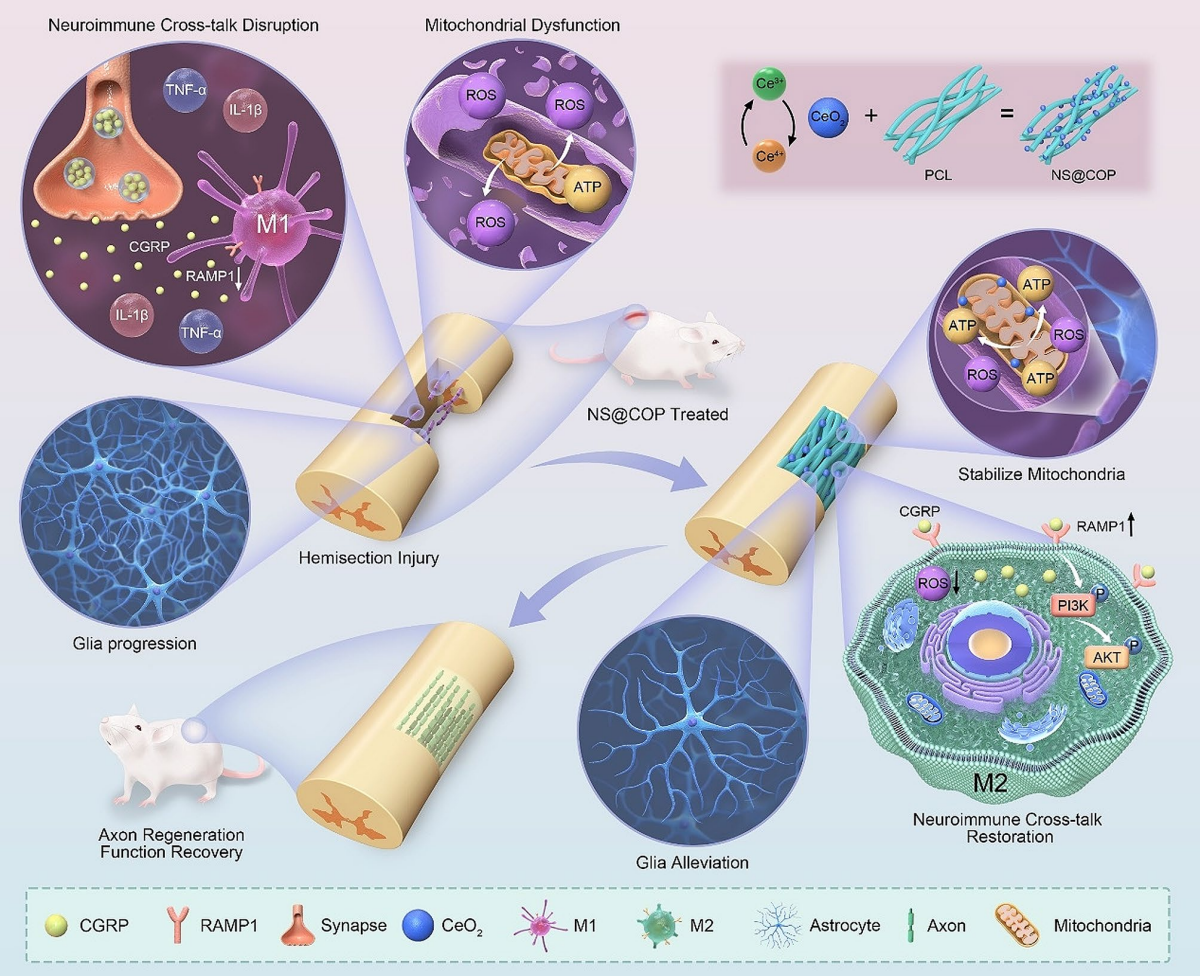
Schematic illustration of the therapeutic nano-enzyme NS@COP scaffold for axon regeneration after spinal cord injury

Here, the experiments of in vitro and in vivo show excellent ROS scavenging ability of NS@COP to address three key challenges that hinder axon repair during SCI (Scheme 1). In in vitro experiment, we applied the H_2_O_2_, CGRP, NS@COP and RAW264.7 co-culture system to conform the neuroimmune cross-talk depend on the CGRP/RAMP1/AKT axis. After implanting NS@COP into the hemi-section SCI rat model, (i) the harsh immune microenvironment, (ii) insufficient energy supply, and (iii) excessive glia scar formation were significantly improved, finally leading to axon repair and functional recovery in vivo. Taken together, our study sheds light on NS@COP could break the vicious circle between sensory nerves and immune cells and mitochondrial function, thereby reducing glial scarring, and facilitating axon regeneration and functional recovery after SCI.

## Materials and methods

### Materials

Poly (caprolactone) (PCL) (Mn average 80 kDa) were purchased from Sigma Aldrich and Cerium oxide nanoparticles (COPs) were purchased from Rhawn Reagent (China). Dimethyl Formamide (DMF) and Dichloromethane(DCM)were purchased from Shanghai Chemical Reagent Corporation, China. The chemicals and reagents were used as the analytical reagents grade.

### Fabrication of CeO_2_ nanoparticle-loaded nanofiber bundle scaffold

The nanofiber scaffold (NS) was fabricated using a dynamic electrospinning machine. Polymer solution with a concentration of 10 wt% was prepared by dissolving PCL into a mixture of DCM and DMF (2:1, v/v), followed by dispersing cerium oxide nanoparticles (COPs) with 0, 0.5, 1, 2, and 4 wt% to acquire different concentrations of electrospinning solution. Then, a 10 mL syringe with a blunt needle (21 G) was loaded with the resultant solution and fed at a rate of 1 mL·h^-1^. A 15 kV voltage was applied to a spinneret 15 cm away from the collecting target. Following the above electrospinning parameters, the collection device is turned into a solid plate to obtain the nanofiber scaffold (NS). All material characterization experiments were performed at Instrumental Analysis Center of SJTU (No. A2021096-1).

### Characterization of CeO_2_ nanoparticle and COPs-loaded nanofiber scaffold

The ultrastructure and nanofiber morphology of freeze-dried scaffold samples were observed by SEM (TM-3000, Hitachi, Japan). The diameters of the nanofibers were calculated using ImageJ software (Bethesda, MD, USA). To investigate the tensile strength of fibrous membrane sections, we cut electrospinning sections into similar dimensions of 1 cm × 1 cm (length × width). The tensile strength of the fabricated fibrous membranes was evaluated with a mechanical machine (Instron 5567, Norwood, MA). In addition, stress-strain curves were established through documentation of load deformation at a speed of 0.5 mm/s. A static contact angle measuring device with DSA 1.8 software (KRUSS, Hamburg, Germany) was used to determine the wettability of the electrospinning membranes. Surface wettability of nanofiber bundle membranes was evaluated by measuring the water contact angle (OCA 15EC, DataPhysics Instrument, Germany). In vitro release was carried out for the COP nanoparticles embedded with nanofiber scaffold. The release profile of COPs from NS@COP was measured by inductively coupled plasma mass spectrometry (ICP-MS, Agilent 7500 series, USA) as previously reported [25]. Briefly, after weighing accurately, the samples were immersed in 10 mL PBS (pH = 7.4), and incubated in a shaker incubator at 100 rpm/min at 37 °C. Fresh PBS was added at regular intervals to replace the solution removed for analysis. At 418 nm, the cerium emission line, the ceria concentration was determined, as well as the accumulated release percentage.

The distribution and diameter of COPs were observed by transmission electron microscopy (TEM, JEM-2100, Japan). The crystal structure of nanoparticles was determined by X-ray powder diffraction (Ragaku Co. Ltd., Tokyo, Japan). XPS (ESCA 2000, Thermo Fisher Scientific, Waltham, MA, USA) was carried out to analyze the chemical ionic status of COPs. The enzyme mimetic activity of COPs was evaluated by. utilizing the total superoxide dismutase assay Kit (Biotech, China) to evaluate the ·O_2_- scavenging activity [26]. 20 µL of COPs dispersion at different concentrations (0, 10, 20, 30, 40 µg/mL) was incubated with assay reagent into a 96-well plate for 30 min. The absorbance at 560 nm was recorded using a Multiskan GO microplate reader (Thermo Fisher Scientific, USA), and calculated the inhibition rate following the manufacturer’s instructions. Then the catalase-mimic activity of COPs was measured with Catalase Assay Kit (Biotech, China) [27]. After drawing the H_2_O_2_ standard curve, a blank control and a sample solution were prepared, and were reacted with the assay agent at 25 °C. Then, a stop and color developing solution were added and incubated at 25 °C for 15 min. The absorbance at 520 nm was measured by a Multiskan GO microplate reader. Finally, the catalase-mimicking activity of COPs was calculated following the manufacturer’s procedures.

### Cell culture, toxicity, and proliferation assay

Isolation and culture of rat cortical neurons: Cortical neurons were isolated and cultured following the established method from the previous study [28]. The cortical neurons were isolated from the fetus of a pregnant E16 rat. Briefly, the cortex was isolated from the Sprague-Dawley rat embryos and rinsed and immersed in Hank’s balanced salt solution at 4 °C. Then the meningeal layer containing vessels was carefully manually stripped, and the cortex tissue was shredded and digested with 0.125% Trypsin-EDTA (Gibco, 25200-056, USA) at 37 °C for 15 min. After incubation for 15 min at 37 °C, the mixed solution was discarded and the remaining samples were rinsed twice in Hank’s solution, and then centrifuged at 1500 rpm/min for 3 min to discard Hank’s solution. The samples were placed in 1 mL cortical neuron culture media containing Neurobasal medium (Gibco, Waltham, MA, USA) supplemented with B27 (Invitrogen Life Technologies, Carlsbad, CA, USA), Gluta-MAX (Invitrogen Life Technologies), and 0.5% penicillin/streptomycin. The pellet was resuspended by triturating about 10 times through 1 mL pipette tips. The single cells were then plated onto 24-well plates at a density of 20 000 cells·cm^–2^ (Fig. S8). The plates were prepared by coating with 20 mg·mL^-1^ poly-d-lysine (Sigma-Aldrich) overnight at 37 °C.

PC-12, DRG, and RAW264.7 cells were purchased from the cell bank of the Chinese Academy of Science (Shanghai, China). Cells were cultured in high-glucose Dulbecco’s modified Eagle’s medium supplemented with 10% fetal bovine serum (Gibco, USA) and 100 U·mL^-1^ penicillin/streptomycin (Servicebio, China). Cells were cultured in 5% CO_2_ incubator at 37 °C. We sterilized NS and other scaffolds by ultraviolet exposure for 4 h. To evaluate the biocompatibility of NS and NS@COP scaffolds to determine their appropriate concentration, we compared 0.5%, 1%, 2–4% COPs in different scaffolds using Live/Dead cell assay kits (Beyotime, China) at 24 h. The samples were incubated with a Calcein-AM and PI (Sigma, Germany) working solution for 20 min in humidified air with 5% CO_2_ at 37 °C. Afterwards, the samples were washed three times with PBS and visualized under a fluorescence microscope (Nikon, Co., Japan). The cell survival rate was calculated using ImageJ software. We also evaluated the cytotoxicity by counting kit 8 (CCK8) assay at 6, 12, and 24 h. Cell proliferation assay was also performed using CCK8 at 24 h, 72 h, and 120 h. The medium from each conduit was evaluated for absorbance at a wavelength of 450 nm and was determined using a multifunctional microplate reader (Thermo Fischer Scientific 3001, USA).

### Scanning electron microscopy and transmission electron microscopy (tem) analysis

For SEM, scaffold samples (1 × 1 mm) were double-fixed with 2.0% glutaraldehyde for 24 h and 2% osmium tetroxide for 2 h. After dehydrating graded ethanol, the critical point dried and sputter-coated with gold for 30 s. The diameter and arrangement of nano fibers were viewed and recorded using SEM (SU8020, Hitachi, Japan). For TEM, tissue samples were fixed overnight in 2.5% glutaraldehyde in 0.2 M phosphate buffer (pH = 7.2). The samples were postfixed with 1% osmium tetroxide (Merck, Germany) for 2 h and dehydrated in an ethanol series: 50, 75, 95, and 100% (×2) for 10 min. Subsequent passages in 100% alcohol and propylene oxide (1:1) and absolute propylene oxide were conducted. Then, the samples were embedded in growing resin (EPON 812, EMS USA) concentrations and placed in a 60 °C oven for 72 h. Finally, 30–50 μm sections were cut with an ultramicrotome, double stained with uranyl acetate-lead citrate, and examined in a transmission electron microscope (TEM, HT7700, Hitachi, Japan), operated at 60 kV. Images were electronically captured by AnalySys 2.0 software and were analyzed individually in Image J software.

### Immunofluorescence (IF) staining of cells

Cell samples were fixed with 4% paraformaldehyde (PFA) for 20 min, and then rinsed three times for 5 min each with phosphate-buffered saline (PBS). The cells were permeabilized in 0.2% Triton X-100 (dissolved in 2% normal goat serum/PBS solution) for 10 min, washed three times in PBS for 5 min, then blocked with 3% normal goat serum/PBS solution for 1 h. Samples were then incubated with primary antibody (rabbit anti-iNOS, 1:200, CST; rabbit anti-Arg1, 1:200, Proteintech; rabbit anti-MAP2, 1:200, Beyotime) overnight at 4 °C, followed by species-specific fluorescent secondary antibody incubation for 2.5 h at room temperature. Nuclei were stained with Dapi for 15 min at room temperature. The images were taken via a fluorescence microscope.

### Detection of ROS level, mitochondria membrane potential

DCFH-DA probe (Beyotime, China) was used to detect ROS level according to instruction. JC-1 (Beyotime, China) and Mito-Tracker Green/Red CmxRos (Thermo Scientific, USA) were used to detect the mitochondrial membrane potential of cells. Briefly, PC-12 cells were cultured in 24-well plates at density of 2 × 10^5^ cells per well in the DMEM complete medium for 4 h. Then the DMEM complete medium was replaced with H_2_O_2_ that was prepared at 500 × 10^− 6^ M in serum-free DMEM for 4 h, the probe was mixed with serum-free culture medium and diluted to work concentration. After incubating for 20 min, the Dapi staining solution (Servicebio, China) was used to stain the nuclei. Then the cell plates were observed and the images were captured using fluorescent microscopy. The cellular mitochondrial membrane potential was determined using JC-1, Mito-Tracker Green/Red CmxRos according to previously mentioned protocol [29].

### Cell culture and treatment

DRG were cultured with H_2_O_2_ (50 × 10^− 6^ M) for 4 h to simulate inflammatory situation. Treatment groups were firstly cultured with H_2_O_2_ (50 × 10^− 6^ M) for 4 h, then changed to normal culture medium for 24 h. The control group was normal cells without any treatment. In order to test whether the CGRP inhibits M1 polarization alternative activation in macrophage, RAW264.7 were firstly treated with H_2_O_2_ (100 × 10^− 6^ M) for 4 h, then replaced the original medium with CGRP (10 nM) and NS@COP, and cultured for 24 h. The procedures of the test whether the RAMP1 is the key modulator to response to CGRP to promoting M2 polarization alternative activation in macrophage were similar to test whether the CGRP inhibits M1 polarization alternative activation in macrophage, except that the BIBN4096s (100nM, 12 h) reprocessing.

### Enzyme-linked immunosorbent assay

The High Sensitivity Enzyme-Linked Immunosorbent Assay Kit of CGRP (Multi Sciences, China) was used according to the manufacturer’s instructions to detect these factors in the DRG co-culture supernatant. The High Sensitivity Enzyme-Linked Immunosorbent Assay Kit of IL-1β, TNF-α, and IL-10 (Multi Sciences, China) were used according to the manufacturer’s instructions to detect these factors in the RAW264.7 cell-culture supernatant.

### Western blotting

Total protein from the RAW 264.7 cells was extracted via cell lysis in RIPA buffer (Beyotime Biotechnology, China) containing 1 mmol/L PMSF. The concentration was determined with a BCA assay. Equal amounts of protein were separated with 10% SDS-PAGE and transferred onto polyvinylidene difluoride membranes (Merck Millipore, Germany). After blocking with blocking buffer (Beyotime, China), the following primary antibodies were incubated at 4 °C overnight: anti-RAMP1 (Abclonal, China, 1:1000), anti-p-AKT (Abcam, USA, 1:1000), anti-AKT (Abcam, USA, 1:1000), iNOS (Abclonal, China, 1:1000), Arg1(Abclonal, China, 1:1000), and anti-β-actin (Beyotime, China, 1:1000). The next day, the membranes were washed with TBST and incubated with horseradish peroxidase (HRP)-conjugated goat anti-rabbit IgG (Proteintech, China, 1:2000) for 1 h at room temperature. The protein signals were detected with enhanced ECL luminescence reagent. The protein levels of the interested proteins were normalized to β-actin and analyzed with Image J software (NIH, Bethesda, MD, USA).

### SCI model in mice and therapy setup

All the procedures regarding the ethics and safety of animal experiments were approved and performed under the guidance of Chang Zheng Hospital of Naval Military Medical University (A2017073). Sprague Dawley rats (200 ± 20 g) (provided by Shanghai SLAC Laboratory Co., Ltd.) were randomly divided into four groups (Control, NS, 0.5%NS@COP, and 1.0%NS@COP) with ten rats per group at each time point. Anesthesia was induced by intramuscular ketamine injection (20 mg/kg). After skin preparation, the back skin was disinfected with povidone-iodine. About 2 cm incision was made longitudinally on the back, and the paraspinal muscles were separated to expose the T9 lamina. The spinal cord was exposed after T9 laminectomy, and then a 1 mm long hemisection defect injury was performed on the right half of the spinal cord with a sharp lancet. The scaffolds were implanted into the defect site. The control group was subjected to injury but received no implantation. The muscle and skin were sutured separately, then the rats were resuscitated in a 37 °C incubator and reared in clean cages at room temperature. Rats were free to access food and water, and the urine was squeezed out every day after the surgery until the urinary function recovered. Upon sacrifice, the repaired tendons were harvested at 4 and 8 weeks for macroscopy, histology, immunofluorescence, TEM images, and ELISA to assess the structural and functional recovery.

### Tissue mRNA expression, determined by RT-PCR after different treatments

Inflammation was investigated with quantitative real-time PCR detection of inflammatory factors. The RT-PCR assays were performed with a PCR system (Applied Biosystems, ViiA™ 7 system) to evaluate the relative IL-1β, TNF-α, IL-4, and IL-10 mRNA levels in lesion site. A housekeeping gene (*GAPDH*) was used as an internal control. The sequences of primers for target genes are presented in Table S1, supplementary information.

### Macroscopic evaluation of repaired spinal cords

At 8 weeks postoperatively, the Achilles tendons were fully exposed and the enough-length spinal cord was harvested. Then, the spinal cords were placed on surgical drapes and photographed by a camera of iPhone.

### Histological staining and biocompatibility evaluation and immunofluorescence

The harvested samples were fixed immediately in 10% formalin for 24 h, and dehydrated with gradient alcohols before embedded in paraffin. Then histological sections were prepared with a microtome with a thickness of 5 μm. A standard procedure for staining soft tissue with H&E was performed to examine the general histological structure of the tissues according to standard procedures. Major functioning organs including heart, liver, spleen, lung, and kidney were also observed by H&E staining to evaluate the biocompatibility of the scaffolds. Immunofluorescence was used to evaluate the effects of NS@COP scaffolds on the protection of spinal cord tissue in vivo. The spinal cord samples were fixed with 4% PFA for 24 h, then dehydrated using 10%, 20%, and 30% sucrose solution sequentially. Then the samples were sliced with a thickness of 10 μm by cryomicrotome (Leica, CM3050S, Germany). The cryosectioned samples were incubated with blocking buffer (0.01 mM PBS, 10% Fetal bovine serum, and 0.3% TritonX-100) for 1 h at room temperature. After that, the sections were incubated with the primary antibody at 4 °C overnight, then incubated with species-specific fluorescent secondary antibody (1:200) for 1 h at room temperature. Nuclei were stained with Dapi for 15 min at room temperature. Lastly, the sections were fixed with an anti-fade mounting medium. The images were taken via a fluorescence microscope. Primary antibodies included: NF-200 (mouse mAb, 1:200, CST, USA), GFAP (rabbit mAb, 1:400, CST, USA), SYP (1:400, CST, USA), MBP (rabbit mAb, 1:200, CST, USA), CD68(mouse mAb,1:100, Santa, USA), CD86 (1:200, Beyotime, china), CD206 (1:200, Beyotime, China). Secondary antibodies included: goat anti-mouse IgG (Alex 488, Proteintech, USA), and goat anti-rabbit IgG (Alex 594, Invitrogen, USA).

### Behavioral evaluation

The recovery of locomotor function of the rats was estimated weekly by using the Basso, Beattie, and Bresnahan (BBB) locomotor rating scale [30]. The rats in all four groups were allowed to move freely in an open area. The full score of the three items was 21 points, which were evaluated by the movements of the hind limbs, the gait and coordination function of the hind limbs, and the fine movements of the claws in the movement. The grades were conducted by two people blindly. Secondly, after the front and rear feet of mice were marked with black and red dyes, they were placed on a track covered with white paper in advance to make the animals run from one end to another. The inter-limb coordination of the center of the front and rear feet of mice on the same side was observed for analysis.

### Statistical analysis

All quantified data were presented as mean ± standard deviation (SD). Statistical analysis was performed using Graph-Pad Prism 7.0 software (USA). One-way or two-way ANOVA coupled with Tukey’s multiple comparison tests were used to evaluate differences between groups. Unpaired two-tailed *t*-test was used to compare two groups. A probability value of *p <* 0.05 was considered statistically significant.

## Results

### Sensory nerve innervation and mitochondrial destruction were observed in the microenvironment of SCI

**Fig. 1:**
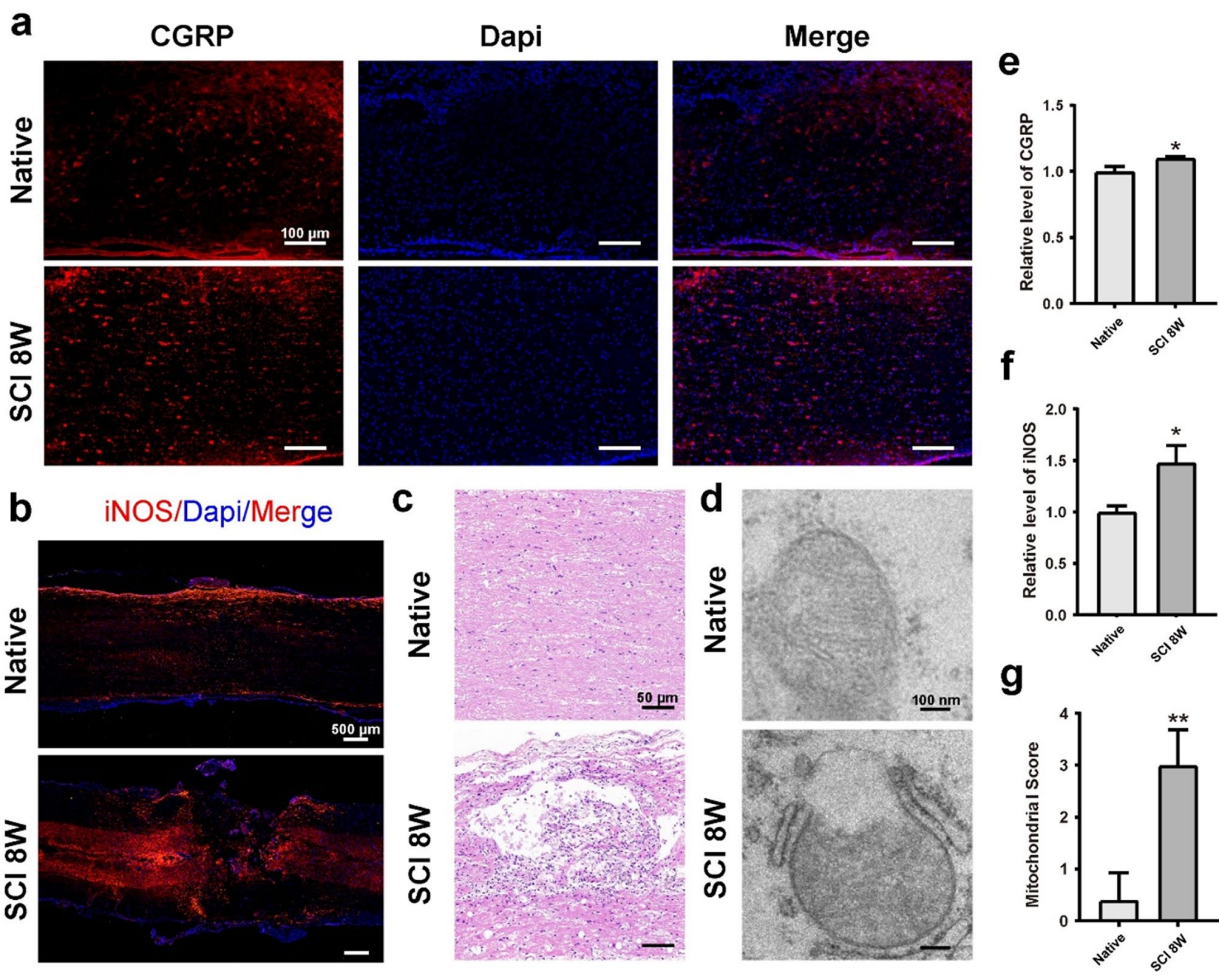
Increased neuropeptide CGRP release, excessive oxidative stress, and mitochondrial structural damage in spinal cord tissue at 8 weeks after injury. **a**) Representative image of immunofluorescence staining of CGRP in native spinal cord and spinal cord at 8 weeks postinjury (*n* = 3). Scale Bar = 100 μm. **b**) Representative image of H&E staining of native spinal cord and spinal cord at 8 weeks postinjury (*n* = 3). Scale Bar = 500 μm. **c**) Representive images of anti-iNOS positive cells in native and injured spinal cord at 8 weeks postinjury (*n* = 3). Scale Bar = 50 μm. **d**) Representive images of mitochondrial structure in native spinal cord and spinal cord at 8 weeks postinjury (*n* = 3). Scale Bar = 100 μm. **e-f**) Quantitative analysis of the relative levels of CGRP and iNOS in native and injured spinal cord at 8 weeks post-injury. **g**) Semiquantitative scoring of mitochondrial structure of native and injured spinal cord at 8 weeks post-injury. Data are presented as mean ± SD. (*) denotes *p* < 0.05, (**) denotes *p* < 0.01, (***) denotes *p* < 0.001, vs. native group

Based on the important role of sensory nerves in modulating the immune microenvironment, we examined how they intervene in areas of SCI lesion and found sensory nerve axons sprouting into the adjacent area of the SCI lesion site, as evidenced by immunofluorescence staining of CGRP, a sensory nerve marker (Fig. 1a). We also observed increased oxidative damage in the injured spinal cord in the SCI group compared to the native spinal cord, as evidenced by the measurement of iNOS levels (Fig. 1, b and f). Oxidative stress causes mitochondrial dysfunction and morphological changes, leading to increased ROS production and mitochondrial destruction [31]. This vicious cycle results in excessive ROS accumulation, triggering secondary damage in SCI [32–35]. The established rat model of hemisected spinal cord defect showed the persistence of structural spinal cord damage at 8 weeks postoperatively (Fig. 1c). Using TEM, we observed that mitochondria in the injured spinal cord were swollen, fissured, and had incomplete outer membranes. The Flameng grading system showed a significantly higher score of spinal cord neuron mitochondria at 8 weeks post-injury compared to controls, indicating severe mitochondrial destruction (Fig. 1, d and g) [36]. The relative CGRP fluorescence intensity was significantly higher in the SCI group compared to the native group at 8 weeks postoperatively (Fig. 1e), suggesting activation of a high sensory innervating program after SCI. These results indicated a coupled process of increased oxidative stress, mitochondrial injury, and sensory nerve innervation. However, the mechanisms by which these coupling phenotypes are involved in the process of SCI repair and how to intervene remains unclear.

### Preparation and characterization of NS@COP nerve scaffold

**Fig. 2:**
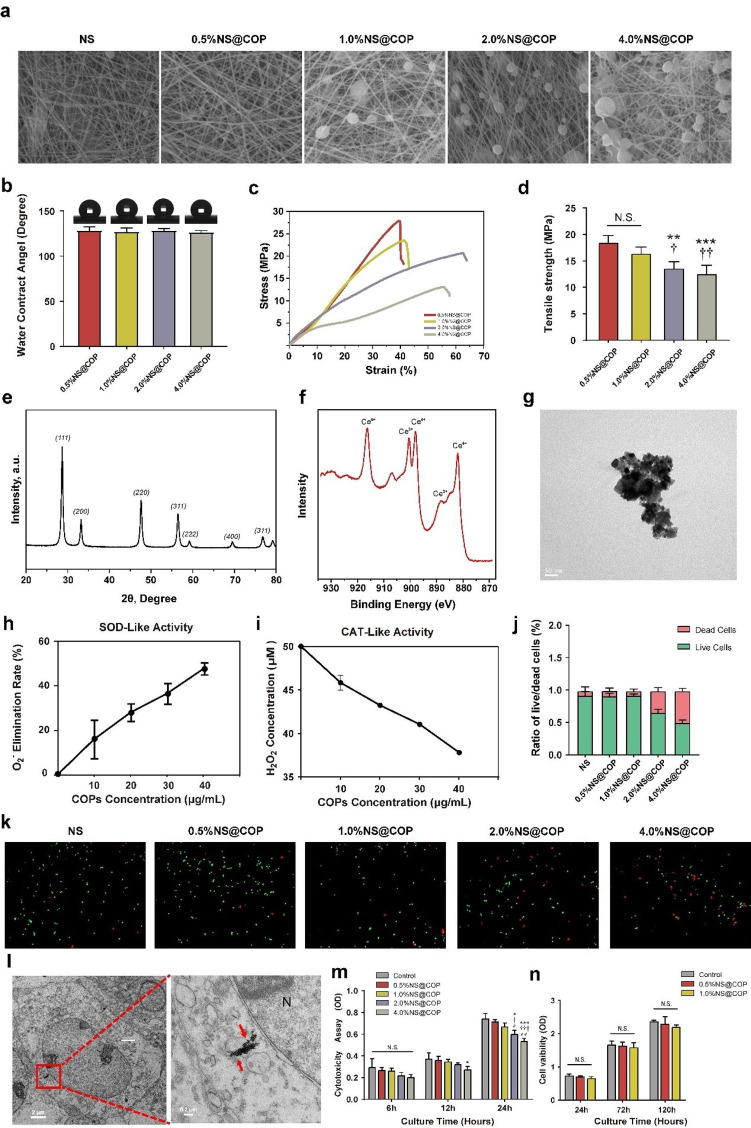
Fabrication, characterization, and cytocompatibility of PCL nanoscaffold with COPs nanozymes. **a**) SEM images of NS and NS@COP scaffolds with different proportions of COP nanoparticles. Scale Bar = 2 μm. **b**) Hydrophilicity of PCL, 0.5%NS@COP, 1.0%NS@COP, 2.0%NS@COP and 4.0%NS@COP (*n* = 5). **c, d**) Strain–stress curve and tensile strength of PCL, 0.5%NS@COP, 1.0%NS@COP, 2.0%NS@COP, and 4.0%NS@COP (*n* = 5). **e, f**, and **g**) XRD, XPS, and TEM analysis of COP nanoparticles (*n* = 5). h, i) SOD and CAT mimic activity of COPs (*n* = 3). **j**) Quantifications of Live/Dead cell staining. **k**) Live/Dead cell staining of PC12 cells co-cultured with scaffolds. Scale Bar = 200 μm. **l**) COPs internalization to cells, visualized by TEM. Red arrows indicate COPs (N: nucleus). Scale Bar = 2 μm. m) Cytocompatibility assay examined at 6, 12, 24 h. o) Cell viability examined at 24, 72, and 120 h. Data are presented as mean ± SD. (*) denotes *p* < 0.05, (**) denotes *p* < 0.01, (***) denotes *p* < 0.001, vs. NS group: (†) denotes *p* < 0.05, (††) denotes *p* < 0.01, (†††) denotes *p* < 0.001, vs. 0.5%NS@COP group: (#) denotes *p* < 0.05, vs. 1.0%NS@COP group

To meet the aforementioned needs, we fabricated a nanoenzyme-based biomimetic nanofibrous scaffold with sustained ROS-scavenging capabilities to aid SCI repair by protecting neuro-mitochondrial dynamics and investigated whether the ROS-scavenging process could coordinate sensory innervation to participate in SCI repair. The resulting single nano-fiber scaffolds were smooth, while the NS@COP fibers had spotted protrusions and rough surfaces. Scanning electron microscopy (SEM) images showed that the NS@COP scaffolds had porous fibers (Fig. 2a). These loose and porous properties facilitated the transport of nutrients between the microenvironment and nerve tissues, providing cues for axon regeneration and pathfinding through the lesion site. The water contact angle of NS showed no significant change after the integration of COPs (Fig. 2b). The stress-strain curves demonstrated that NS and different NS@COPs had similar tensile strength moduli, providing stable mechanical support for early-stage spinal cord repair (Fig. 2, c and d). XRD analysis confirmed that COPs had a typical fluorite cubic structure (Fig. 2e). X-ray photoelectron spectroscopy (XPS) analysis demonstrated the chemical bonding peaks related to Ce^3+^ and Ce^4+^ at the corresponding binding energies, indicating the favorable biological ROS scavenging properties due to self-redox reactions (Fig. 2f) [21, 37, 38]. Furthermore, transmission electron microscopy (TEM) images revealed the typical morphology of COPs, with a dotted crystal pattern and an average size of 20.0 nm (Fig. 2g). We also measured the enzyme-mimic activity of COPs, including SOD and CAT (Fig. 2, h and i), confirming their ability to catalyze superoxide anions and hydrogen peroxide in a concentration-dependent manner and demonstrating their high ROS scavenging capability [39].

To determine the appropriate concentration of NS@COP for neural growth, we evaluated their biocompatibility using Live/Dead cell kits and cell counting kit 8 (CCK8) assay. We found that 1% NS@COP was much less cytotoxic than 2% and 4%, and slightly more toxic than the 0.5% counterpart after 24 h (Fig. 2, j, k and m). Thus, we selected 0.5% and 1% NS@COP for the following studies. TEM ultrastructural images showed that the released COPs were well internalized into the cytoplasm of neurons (Fig. 2l), suggesting their potential to inhibit excessive intracellular ROS and promote repair in injured neuron cells. The cell proliferation assay using CCK8 at 24, 72, and 120 h showed no significant differences in cell proliferation among NS, 0.5% NS@COP, and 1% NS@COP (Fig. 2n). Overall, we successfully engineered an enzyme-mimicking, energy-supporting COPs-integrated NS scaffold that provides adequate physical and biochemical cues for maintaining energy homeostasis and restoring redox microenvironment in spinal cord repair.

### NS@COP enhances sensory nerve-regulated macrophage fate determination toward an M2 anti-inflammatory phenotype via unmasking CGRP/RAMP1/AKT axis

Previous studies have shown that sensory nerves play a crucial role in maintaining tissue homeostasis and promoting tissue regeneration by secreting sensory neuropeptide calcitonin gene-related peptide (CGRP), which is known to modulate anti-inflammatory macrophage function [14, 15, 40, 41]. In addition, the receptor for CGRP, formed by RAMP1 and its co-receptor, is highly expressed on the macrophage, allowing then to respond to modulation by sensory nerve. On the other hand, ROS-induced aberrant macrophage fate determination toward a pro-fibrotic phenotype can promote glial scar formation and hinder SCI functional recovery [7, 8]. Therefore, we hypothesized that ROS may block the pro-regenerative role of sensory nerves in macrophage communication after SCI via CGRP/RAMP1 axis.

**Fig. 3:**
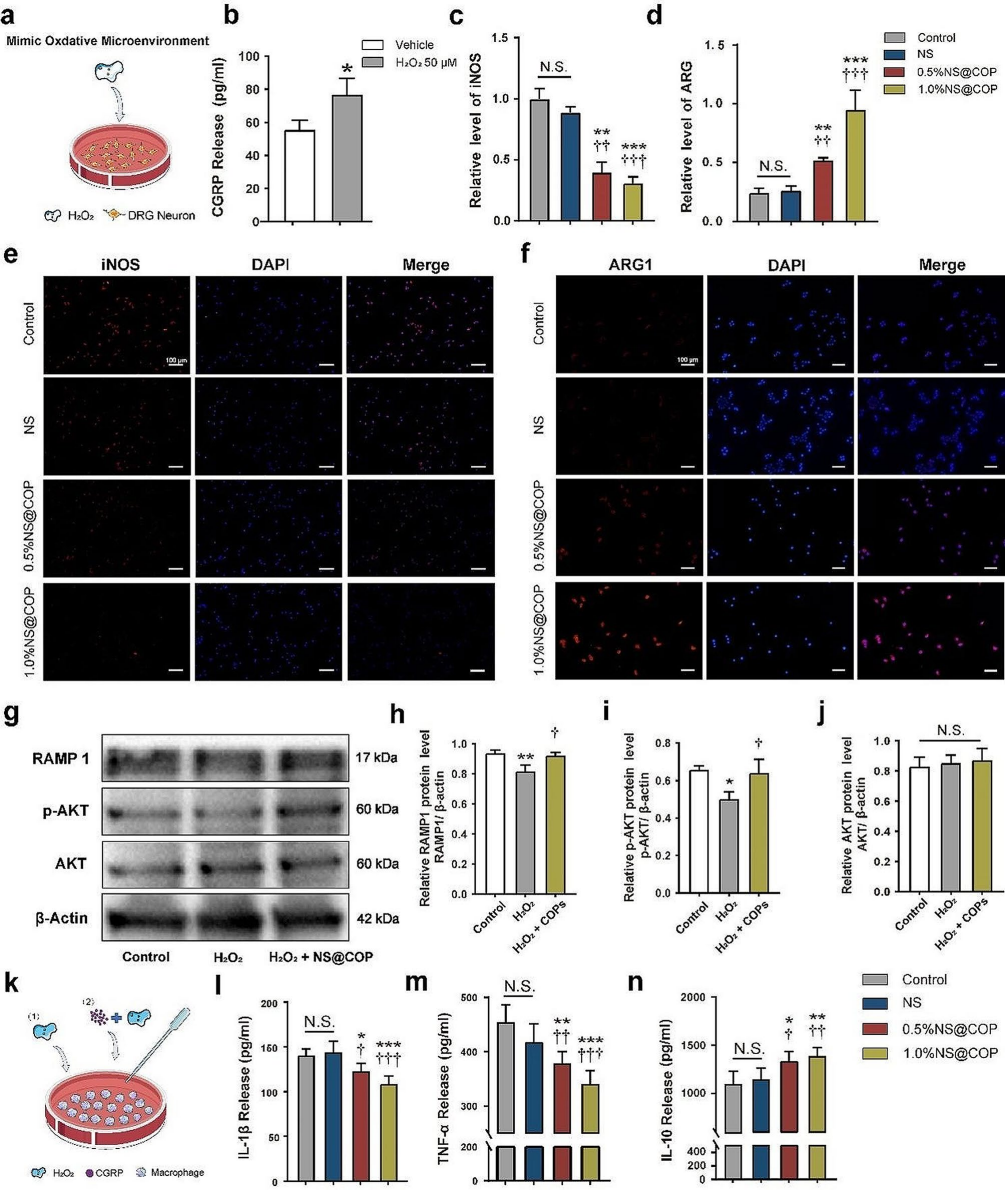
Regulation of macrophage anti-inflammatory activity by the NS@COP scaffold. **a-b**) CGRP secretion was detected by ELISA. **c-d**) Quantitative analysis of the relative levels of iNOS and ARG1 in control, NS, 0.5%NS@COP, and 1.0%NS@COP group. **e-f**) Representative images of immunofluorescence staining of iNOS and ARG1 in RAW264.7 cells stimulated by H_2_O_2_ (100 µM) and treated with CGRP (10 nM). Scale Bar = 100 μm. (**g**) Western blot analysis of RAMP1, p-AKT, AKT, and β-actin expression in RAW264.7 cells with different treatments. (*n* = 3). (**h–j**) The results of western blot analysis are expressed as percentages of positive mean ± SD. (*n* = 3). **k**) Schematic representation of the RAW264.7 macrophage with different treatments. (l-n) Release of IL-1β, TNF-α, and IL-10 protein was tested by ELISA after the indicated periods. (*) denotes *p* < 0.05, (**) denotes *p* < 0.01, (***) denotes *p* < 0.001, vs. control group: (†) denotes *p* < 0.05, (††) denotes *p* < 0.01, (†††) denotes *p* < 0.001, vs. NS group

To investigate the hypothesis, we measured the secretion of CGRP in DRG neurons using ELISA and found H_2_O_2_ (50 µM) significantly increased the expression of CGRP protein in DRG neurons (Fig. 3a and b). The results were consistent with previous studies [42]. To confirm the influence of CGRP on macrophage fate determination under varying oxidative conditions, we treated RAW264.7 macrophages with CGRP and H_2_O_2_ (Fig. 3k). We found that CGRP significantly reduced the expression of iNOS protein, an M1 macrophage marker, as well as the secretion of IL-1β and TNF-α in macrophages primed by NS@COP (Fig. 3, c, e, l, and m). Furthermore, CGRP significantly increased the expression of Arg1 protein, an M2 macrophage marker, and the secretion of IL-10, a potent anti-inflammatory cytokine (Fig. 3, d, f and n). These data suggest that CGRP could direct macrophage fate toward an M2 anti-inflammatory phenotype. However, this does not explain why increased sensory innervation fail to coordinate sufficient M2 anti-inflammatory macrophage commitment to reduce glial scar formation. To address this issue, we further examined the downstream effector of CGRP in macrophages. Surprisingly, we discovered that the expression of CGRP receptor activity modifying protein 1 (RAMP1) was significantly downregulated in H_2_O_2_-stimulated macrophages, and this effect was abolished by NS@COP application (Fig. 3, g-h). Next, we pretreated macrophage with BIBN4096s, an antagonist of RAMP1 signaling [43]. Pre-treatment with BIBN4096s significantly reduced the expression of RAMP1 and Arg1 (anti-inflammatory macrophage marker) and increased the expression of iNOS (pro-inflammatory macrophage marker) compared NS@COP group (Fig. S1-2). These results suggest that reduced responsiveness of macrophages to CGRP signals under oxidative stress is responsible for the attenuated transition of macrophage towards the M2 phenotype during SCI repair, and that cleavage of ROS by NS@COP application could rescue this pathologic process.

The PI3K/AKT pathway plays a crucial role in the survival, proliferation, and migration of macrophages. Changes in AKT modulation of the AKT activity levels in macrophages significantly affect their polarization phenotype [44]. Then, we quantified the AKT signaling proteins by Western blotting and observed a significant increase in p-AKT protein level in the NS@COP-treated RAW264.7 macrophage cells (Fig. 3i), but no significant difference in Akt protein level among groups (Fig. 3j). Taken together, this evidence suggests that NS@COP could scavenge ROS to simultaneously increase the RAMP1 expression in macrophages, enabling efficient CGRP/RAMP1/AKT signaling flow to regulate macrophage fate determination.

### NS@COP maintains mitochondrial ΔΨm to enable sufficient energy supply to cells

**Fig. 4:**
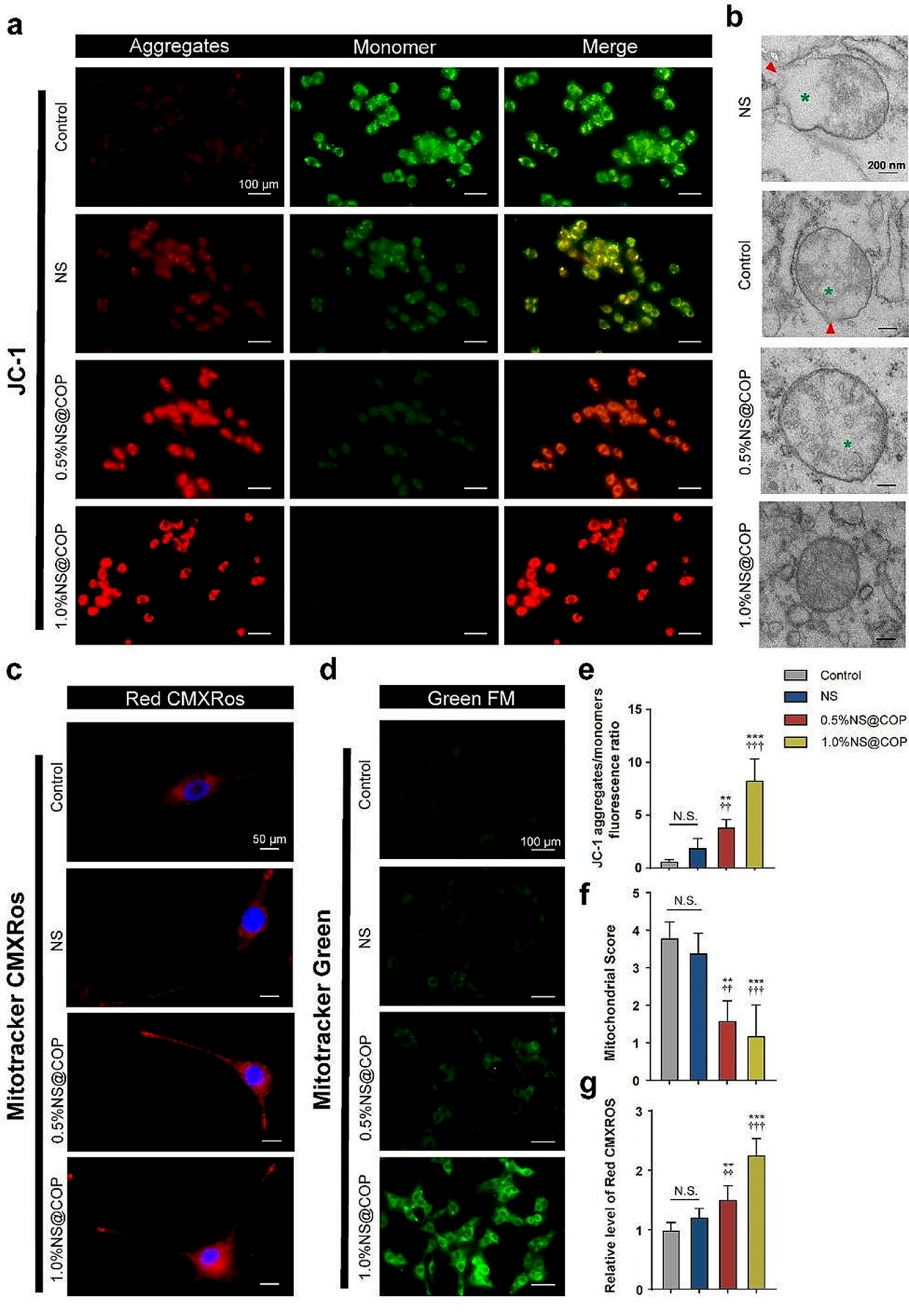
NS@COP restored mitochondrial membrane potential and ultra structure of cells in vitro. **a**, **c**, and **d**) Representative images of immunofluorescence staining of mitochondrial membrane potential (ΔΨm) detected by JC-1 probe and Mitotracker indicator, respectively. **b**) Representative TEM images of mitochondrial ultrastructure of cells stimulated by H_2_O_2_ (100 µM) and treated with different NS@COP for 24 h (*n* = 3). Scale Bar = 200 nm. g, h) Quantitative analysis of the relative fluorescent level of (**a**) and (**c**) (*n* = 3). **f**) Semiquantitative scoring of mitochondrial structure of control, NS, 0.5%NS@COP, and 1.0%NS@COP group, respectively (*n* = 3). Data are presented as mean ± SD. (*) denotes *p* < 0.05, (**) denotes *p* < 0.01, (***) denotes *p* < 0.001, vs. control group: (†) denotes *p* < 0.05, (††) denotes *p* < 0.01, (†††) denotes *p* < 0.001, vs. NS group

ROS is a double-edged sword, playing various significant roles in modulating the mitochondrial function under different conditions. Under physiological conditions, ROS is generated by the cellular respiratory process of mitochondria, and the balance between ROS generation and internal ROS cleavage systems maintains normal cell metabolism [45]. However, under SCI condition, the accumulation of ROS and impaired ROS cleavage capability disrupt this balance, leading to mitochondrial structural damage and reduced energy supply to neurons, ultimately impairing axon growth [12, 46–48]. We conducted a series of experiments to demonstrate the oxidative stress scavenging and enzyme-like antioxidant ability of NS@COP (Fig. S4). First, we tracked the presence of COPs released from scaffold in cultured cells using TEM. After endocytosis, the COPs were primarily present at two locations: the mitochondrial outer membrane and inner leaflet of the plasma membrane in consistent with previous report [21] (Fig. S4). To investigate the effect of NS@COP to counteract the attenuation of neuronal energy supply induced by ROS, we measured mitochondrial membrane potential (ΔΨm), an early marker of cellular apoptosis that reflects the state of the mitochondria [49, 50]. Using the JC- 1 probe, we found that the ΔΨm of mitochondria in cells co-treated with NS@COP was significantly higher than in the control group (Fig. 4a). In addition, ultrastructural changes in mitochondria closely reflect functional changes, such as the intact outer membrane, which is one of the most important indicators of the full function of the oxidative respiratory chain that supplies energy [51]. We further observed mitochondrial ultrastructure using TEM and found that the mitochondria of spinal cord neurons in the control group had a ruptured outer membrane, while the mitochondria in the NS@COP group had an intact outer membrane and unswollen cell body, exhibiting the lowest mitochondrial score among the four groups (Fig. 4b). Two other fluorescent indicators of ΔΨm, Mito-tracker RED and Mito-tracker Green, also confirmed changes in mitochondrial membrane potential (Fig. 4, c and d). These results suggest that NS@COP has a strong ability to maintain normal mitochondrial ultrastructure and membrane potential, effectively scavenging ROS to stabilize mitochondrial membrane potential and aid neuronal energy supply.

#### NS@COP promotes cortical neuron axon regeneration under oxidative stress

**Fig. 5:**
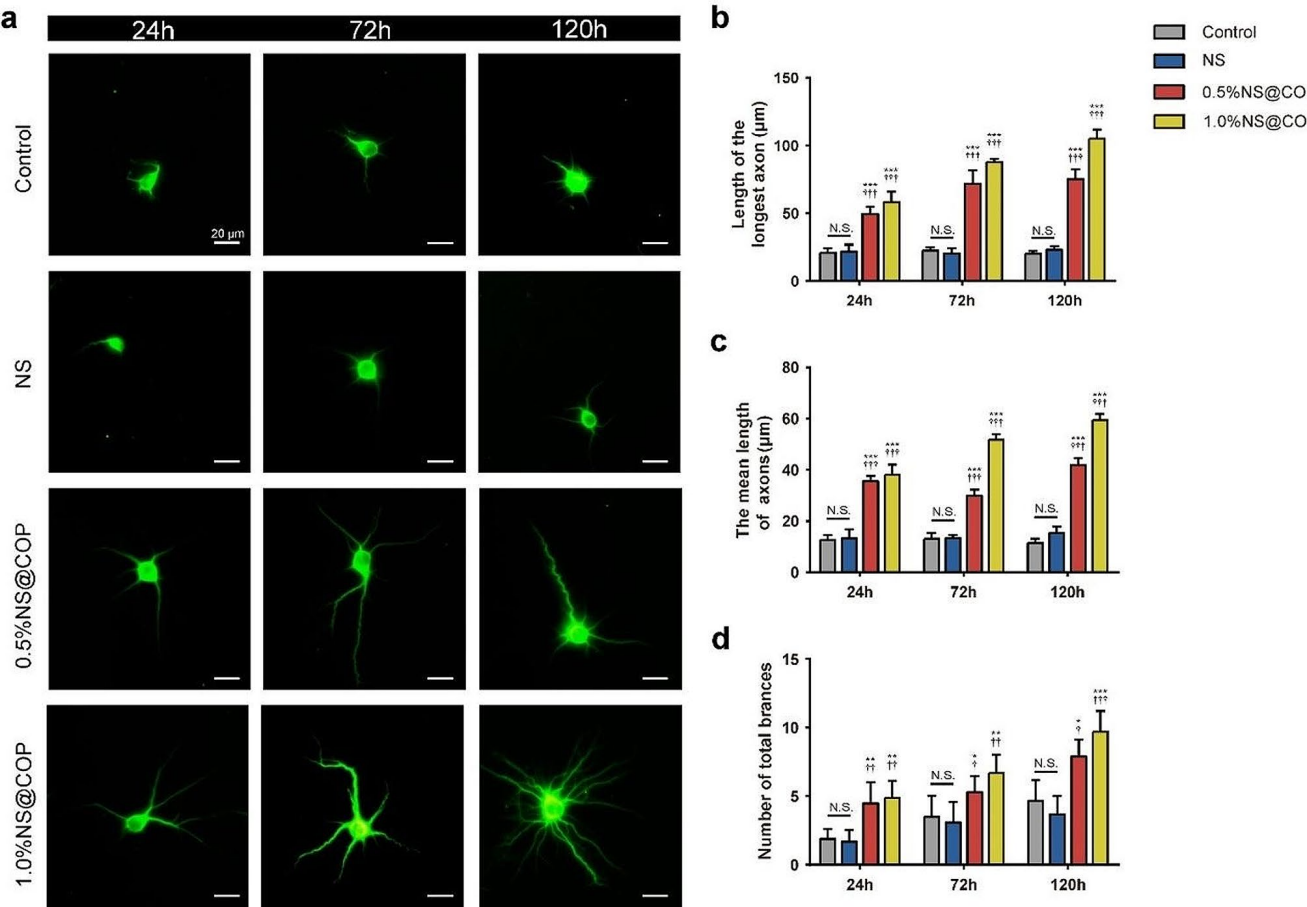
The NS@COP scaffold promotes axon outgrowth of H_2_O_2_-modeled cortical neurons in vitro. **a**) Representative images of immunofluorescence staining of MAP2 of cortical neurons co-cultured with different NS@COP scaffolds for 24 h, 72 h, and 120 h, respectively (*n* = 3). Scale Bar = 20 μm. **b-d**) Quantitative analysis of length of the longest axon, the mean length of the axons, and the number of total branches for 24 h, 72 h, and 120 h, respectively (*n* = 3). Data are presented as mean ± SD. (*) denotes *p* < 0.05, (**) denotes *p* < 0.01, (***) denotes *p* < 0.001, vs. control group: (†) denotes *p* < 0.05, (††) denotes *p* < 0.01, (†††) denotes *p* < 0.001, vs. NS group

Previous studies have shown that ROS impairs the regeneration of neuronal axons, leading to reduced functional recovery after SCI [4–6]. In addition to its immune modulatory effects, we explored the potential of NS@COP to promote neuronal axon regeneration under oxidative stress. To this end, we examined the ability of NS@COP to protect cortical neurons from H_2_O_2_-induced damage and restore dendritic sprouting and axonal growth. We quantified the length and number of branching neurites at predetermined time points (Fig. 5a) and found that the NS@COP group had a significantly longer length of the longest axon and more branches than the H_2_O_2_-treated group (Fig. 5b). Additionally, the mean length of branches was significantly higher in the 1%NS@COP group than in the other groups (Fig. 5, c and d), indicating that NS@COP promotes branch formation and neuronal axon outgrowth, thereby protecting neurons against ROS-induced damage.

### NS@COPs effectively promote the recovery of locomotor functions in a rat hemisecting SCI model

**Fig. 6:**
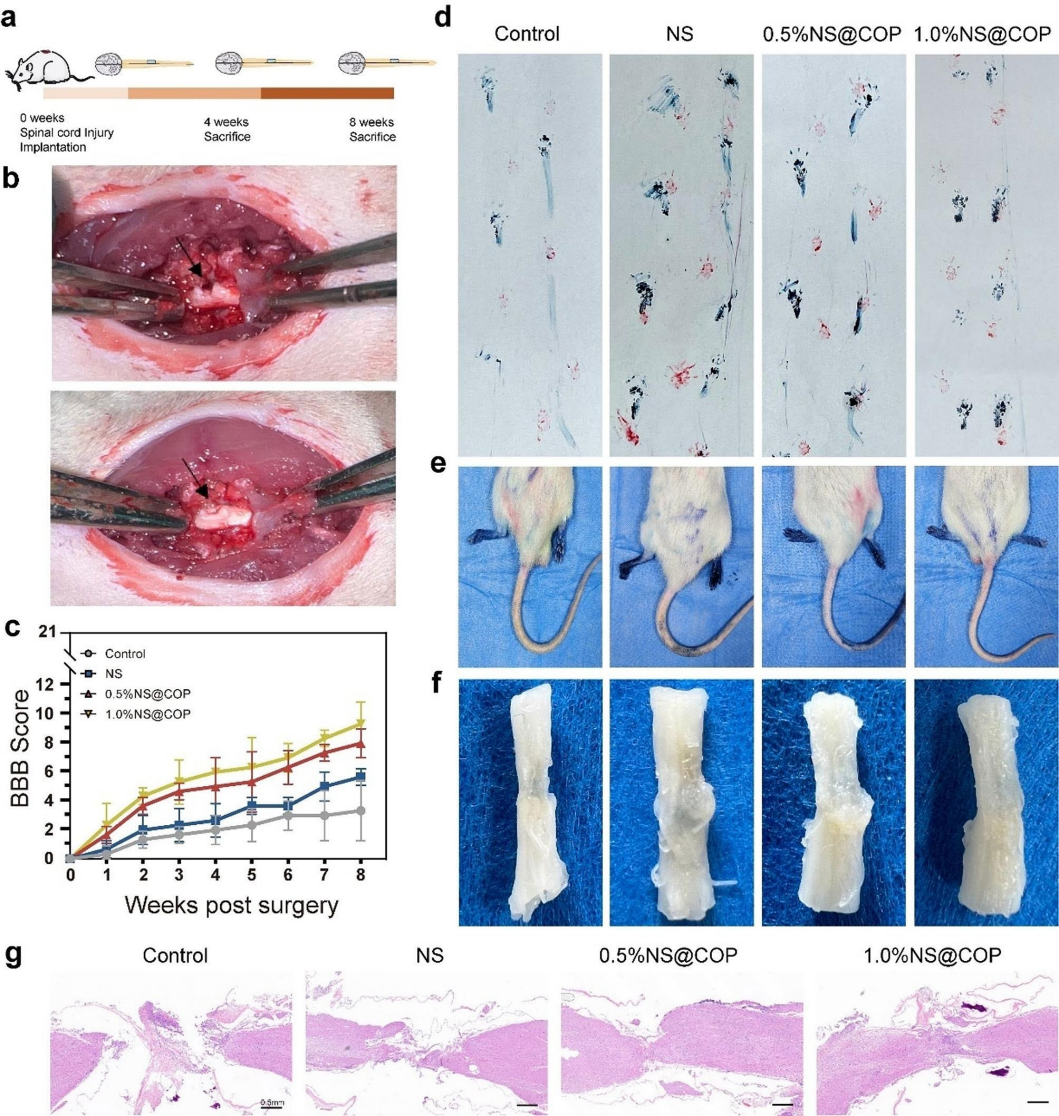
Motor function recovery in spinal cord injury rats after implantation of NS@COPs. **a**) The experimental timeline schematics. **b**) Images of implanting scaffolds during SCI operation (Black arrow ahead) points to the surgical sites. **c**) Function recovery of rats on week 8 post-surgery evaluated by BBB scores. **d**) Representative footprints of the rats in the control, NS, 0.5%NS@COP, and 1.0%NS@COP groups. The fore-and hind limbs of the rats were inked red and blue, respectively. **e**) Typical hindlimbs-spreading status of SCI rats on week 8 post-surgery. **f**) Representative images of macrography of spinal cord operative segments at 8 weeks post-surgery. **g**) Histological examination on the longitudinal sections of the spinal cords collected on week 8 post-surgery. *n* = 3. Scale Bar = 0.5 mm

The experimental timeline schematic is presented in Fig. 6a, and the surgical procedure is shown in Fig. 6b. We employed the Basso, Beattie, and Bresnahan scale (BBB) to assess the degree of hindlimb locomotion functional recovery after SCI. The right hindlimbs of rats in the control group exhibited almost total paralysis without treatment, with BBB scores of no more than eight weeks after SCI. In contrast, rats in the 1% NS@COP group showed significantly higher BBB scores (9.33 ± 1.25) compared to the NS group (5.67 ± 0.47) and the SCI group (3.33 ± 1.70) at 8 weeks post-surgery, indicating effective restoration of hindlimb function (Fig. 6c). To evaluate the effect of NS@COP on locomotor recovery, we conducted a footstep imprinting experiment 8 weeks post-injury. We observed that hind paw footprints (blue ink) in the NS@COP group largely overlapped with the forepaw footprints (red ink), indicating improved coordination between the forepaws and hind paws during walking. In contrast, the control group exhibited severe footstep disorder with a wave shape and dragged movement, as the hindlimbs of SCI rats were unable to support their weight (Fig. 6d). We also checked the toe spreading of hind paws with different scaffolds (Fig. 6e). After observing the gross tissue, we found that visible cavities remained at the lesion sites in the control group, while these defects were significantly reduced in the 1%NS@COP group (Fig. 6f), suggesting efficient neuronal regeneration inside the NS@COP scaffold.

To visualize the recovery of spinal cord tissue, we harvested tissues while preserving 1 cm of normal tissue at both ends of the lesion site. H&E staining and tissue morphology in the control group revealed severe tissue damage and significant cystic cavitation, whereas rats in the 0.5%NS@COP and 1.0%NS@COP groups showed reduced lesion area and better tissue reconnection (Fig. 6g). Notably, implantation of 1.0%NS@COP significantly accelerated spinal cord healing compared with other groups. In summary, our results demonstrate that NS@COP treatment significantly enhances the behavioral function of rats after SCI, as evidenced by improved hindlimb coordination and functional recovery, and efficient neuronal tissue regeneration.

We also verified the satisfactory biocompatibility of NS and NS@COP scaffolds in vivo. The biocompatibility of nanomaterials is a critical concern, as they may release toxic compounds and accumulate in organs when the substrate degrades. The primary function organs, including the heart, liver, lung, spleen, and kidney, exhibited no structural abnormalities at 8 weeks post-implantation of different scaffolds (Fig. S7). Therefore, our NS@COP scaffold can function as a bio-friendly and superior bioactive nanomaterial for axon regeneration after severe spinal cord injury.

#### NS@COP restores blocked sensory nerve to macrophage communication to decrease glia scar formation

**Fig. 7:**
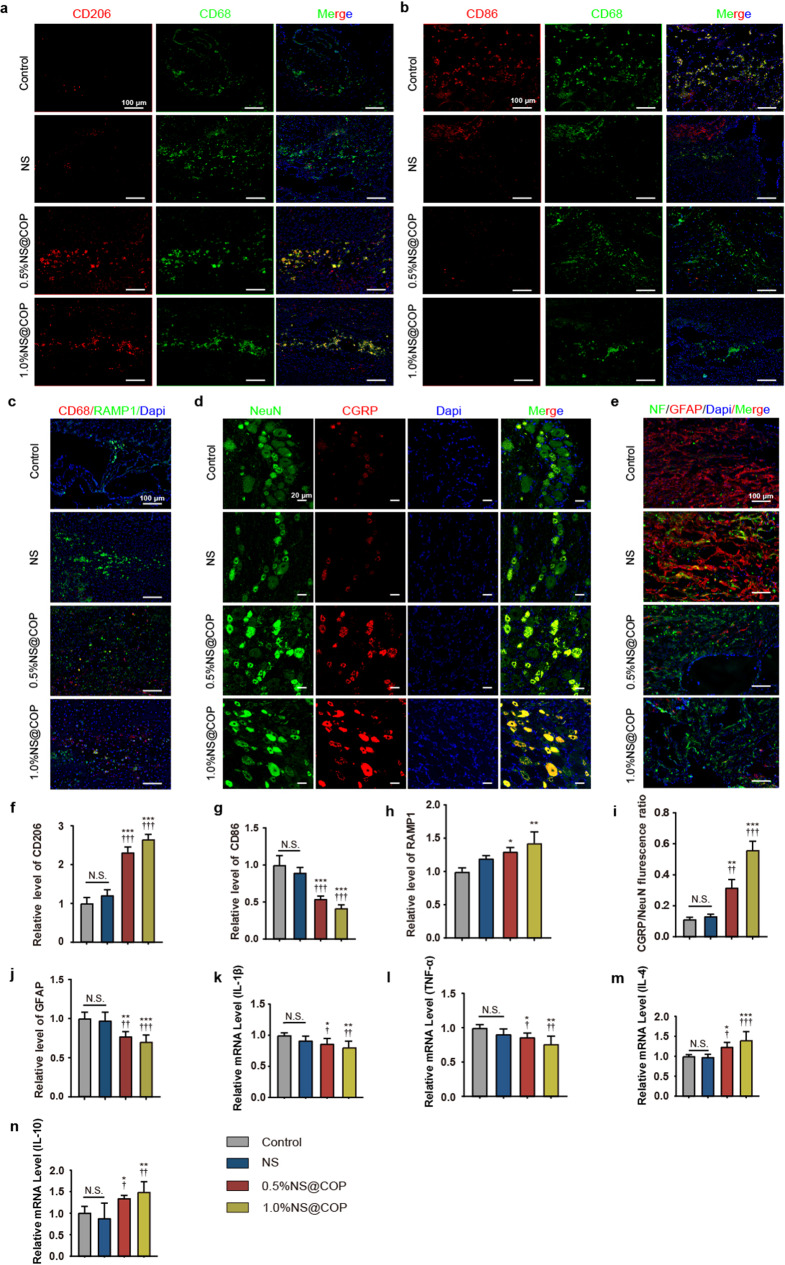
Implantation of the NS@COP scaffold enhances sensory neuropeptide production, promoting the transition of M1 to M2 macrophages and reducing inflammation in rats with spinal cord injury. **a-b**) Representative images of immunofluorescence staining of CD86 and CD206 of spinal cord tissue in control, NS, 0.5%NS@COP and 1.0%NS@COP groups at 8 weeks postsurgery (*n* = 5). Scale Bar = 100 μm. **c**) Representative images of immunofluorescence staining of RAMP1 of spinal cord tissue in control, NS, 0.5%NS@COP, and 1.0%NS@COP groups at 8 weeks post surgery (*n* = 5). Scale Bar = 100 μm. **d**) Representative double-immunofluorescence images of DRG neurons, in which neurogenic peptide and stimulation are characterized by the presence of CGRP, and the neurons are indicated by NeuN (*n* = 5). Scale Bar = 20 μm. **e**) Representative images of immunofluorescence staining of the injured spinal cord segments. Spinal cord lesion sites were visualized using NF200 and GFAP staining (*n* = 5). Scale Bar = 100 μm. **f**) Semiquantitative analysis of the relative fluorescent intensity of (**c**). **g-i**) Semiquantitative analysis of the relative fluorescent intensity of (**a**), (**b**), and (**e**). Data represented as mean ± SD. j-m) Levels of mRNA for IL-1β, TNF-α, IL-4 and IL-10 (*n* = 5). (*) denotes *p* < 0.05, (**) denotes *p* < 0.01, (***) denotes *p* < 0.001, vs. control group: (†) denotes *p* < 0.05, (††) denotes *p* < 0.01, (†††) denotes *p* < 0.001, vs. NS group

The normal commitment of macrophage fate from M1 to M2 is essential for shifting the microenvironment from a pro-inflammatory to an anti-inflammatory state during SCI repair. This is a critical step to remove the local inflammatory microenvironment and rehabilitate the regenerative potential of surrounding neurons. However, excessive ROS accumulation has been shown to impair macrophage fate determination and delay the onset of the anti-inflammatory phase, which may lead to excessive formation of glial scars and impair functional recovery after SCI [9–12]. Therefore, we examined macrophage fate determination in vivo by immunostaining for expression of CD86 (an M1 macrophage marker) and CD206 (an M2 macrophage marker). After 1% NS@COP treatment, the CD206 signal increased while the CD86 signal decreased (Fig. 7, a, b and Fig. S5, a, b). We also found that the expression level of RAMP1 in macrophage significantly increased in NS@COP group, compared with control group (Fig. 7, c, h and Fig. S5c). To validate the mechanism of ROS blockade on sensory nerve-regulated macrophage fate and the formation of glial scars in vivo, we assessed the effect of implantation of NS@COP on sensory innervation during SCI repair. In addition, we isolated DRG adjacent to the lesion site and found a significant increase percentage of CGRP-positive DRG neurons in the 1% NS@COP group (Fig. 7, d and i). The above data suggested that the ROS scavenging could modulate the effect of CGRP on the conversion of macrophage fate from M1 to M2 type.

Activated astrocytes contribute to the formation of glial scars, which act as a physical barrier to neural regeneration and axonal pathfinding through the lesion area, ultimately leading to limited functional recovery after SCI [2, 3]. In the control group, massive astrocytic glial scars formed hypertrophic processes that densely overlapped and packed around the lesion area, with very few scattered axons at the epicenter of the lesion. We found that glial scar formation was significantly attenuated in the 1% NS@COP group compared with controls (Fig. 7, e and j).

We also measured the mRNA expression of macrophage markers, including IL-1β, TNF-α, IL-4, and IL10, using RT-PCR analysis. Gene expression analysis showed that mRNA levels of the anti-inflammatory cytokines IL-4 and IL10 were significantly higher in the 1% NS@COP group than in the control group. The mRNA expression levels of the proinflammatory cytokines IL-1β and TNF-α in the 1% NS@COP group were significantly lower than those in the control group (Fig. 7, k-n). Both immunofluorescence and gene expression analysis indicated a significant increase in anti-inflammatory factors in the 1% NS@COP group. These results suggest that effective cleavage of ROS by NS@COP is essential for restoring and amplifying the effect of sensory nerve-regulated macrophage M2 commitment to reduce glial scar formation and aid SCI repair.

#### NS@COPs efficiently restore mitochondrial structural integrity in spinal cord neurons after SCI

**Fig. 8:**
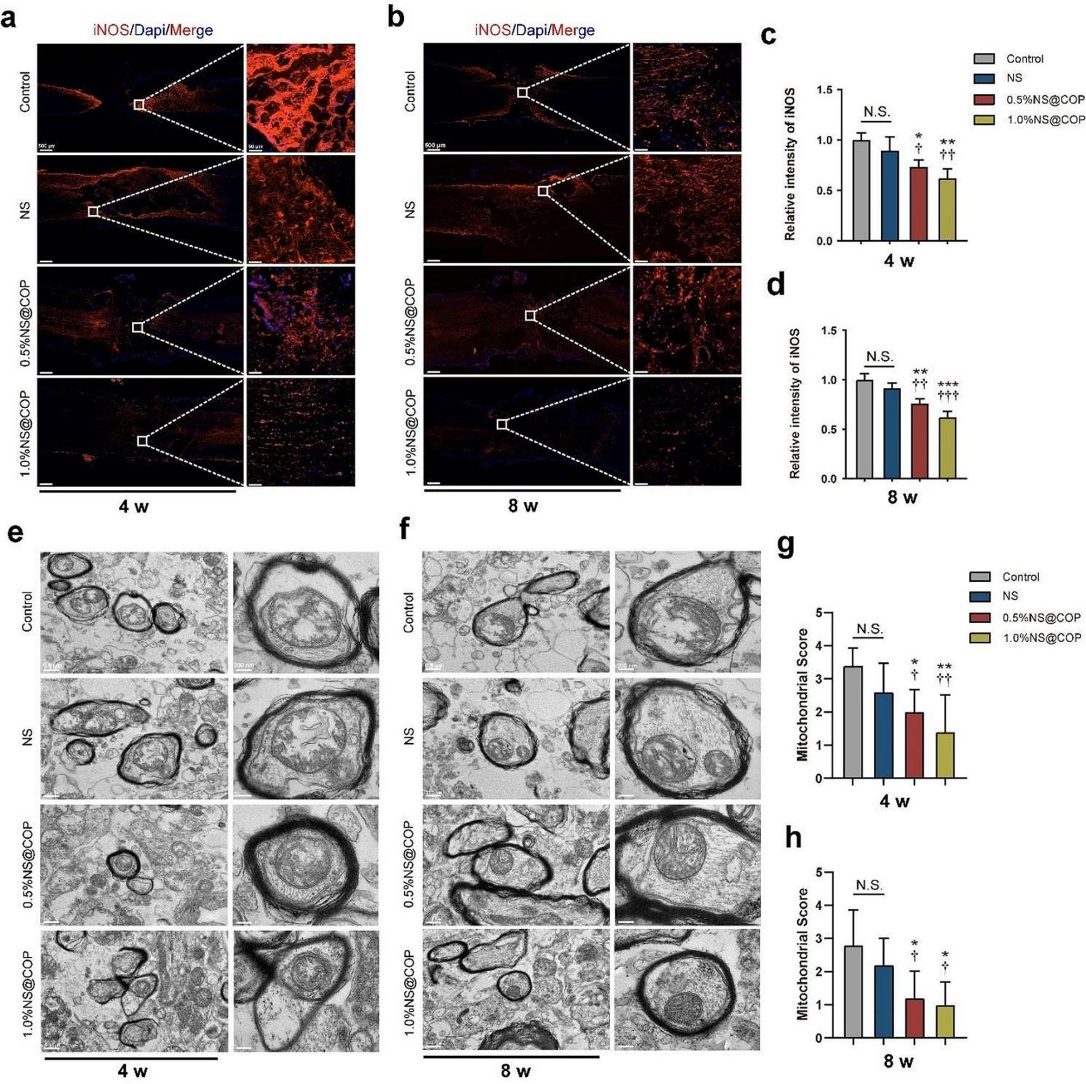
NS@COP alleviated the oxidative damage and restored mitochondrial ultrastructure at predetermined time post-spinal cord injury. a-b) Representative images of immunofluorescence staining of iNOS of spinal cord surgery segments from different groups at 4 and 8 weeks postinjury. Scale Bar = 500 μm. The magnified images on the right are from the box area of the image on the left (*n* = 3). **c-d**) Quantitative analysis of the relative fluorescent intensity of (**a**) and (**b**) (*n* = 3). **e**) Representative TEM images of mitochondrial ultrastructure of axons at 4 weeks postoperatively. Scale Bar = 0.5 μm. **f**) Representative TEM images of mitochondrial ultrastructure of axons at 8 weeks postoperatively. Scale Bar = 0.5 μm. **g-h**) Semiquantitative scoring of mitochondrial structure of 4 and 8 weeks postoperatively (*n* = 5). Data are presented as mean ± SD. (*) denotes *p* < 0.05, (**) denotes *p* < 0.01, (***) denotes *p* < 0.001, vs. control group: (†) denotes *p* < 0.05, (††) denotes *p* < 0.01, (†††) denotes *p* < 0.001, vs. NS group

To investigate the effects of NS@COP on spinal cord neuronal energy metabolism in vivo, we established a hemisected model using previously reported procedures [52]. First, we assessed the ability of NS@COP to inhibit excessive oxidative stress and protect mitochondria. We measured the expression level of iNOS at the lesion site and found that at 8 weeks post-SCI, a large number of cells in the control group stained positive for iNOS. However, in the 1% NS@COP group, the number of positive cells and fluorescence intensity were significantly reduced, indicating effective ROS scavenging activity enabled by NS@COP implantation (Fig. 8, a and b). Semi-quantitative analysis revealed that iNOS expression in the 1% NS@COP group was reduced to approximately 62% of that in the control group (Fig. 8c). While the average reduced intensity of iNOS was also detected in the control group at 8 weeks, significant differences were still observed between the 1% NS@COP group and the control group (Fig. 8d). Notably, NS@COP implantation alleviated oxidative damage postoperatively, breaking the vicious cycle of spinal cord damage.

To assess mitochondrial morphology damage, we used TEM to visualize mitochondrial ultrastructure and calculated a mitochondrial score. The mitochondrial score of the 1% NS@COP group was significantly lower than that of the control group and NS group at 4 weeks postoperatively (Fig. 8, e and g), suggesting that NS@COP application is capable of preserving the structural integrity of mitochondria under oxidative stress. Intriguingly, at 8 weeks postoperatively, part of the mitochondria in the 1% NS@COP group returned to nearly normal morphology, without swelling or membrane distortion, exhibiting the lowest average score among the groups (Fig. 8, f and h). This indicates that sustained ROS cleavage by NS@COP implantation is pivotal for restoring normal cell energy metabolism. Overall, our NS@COP treatment reduced oxidative damage in nervous tissue, improved antioxidant enzyme activities, and promoted mitochondrial structure recovery at 4 and 8 weeks after SCI.

#### NS@COPs reduce injury-related cavity size and promote axon regeneration after SCI

**Fig. 9:**
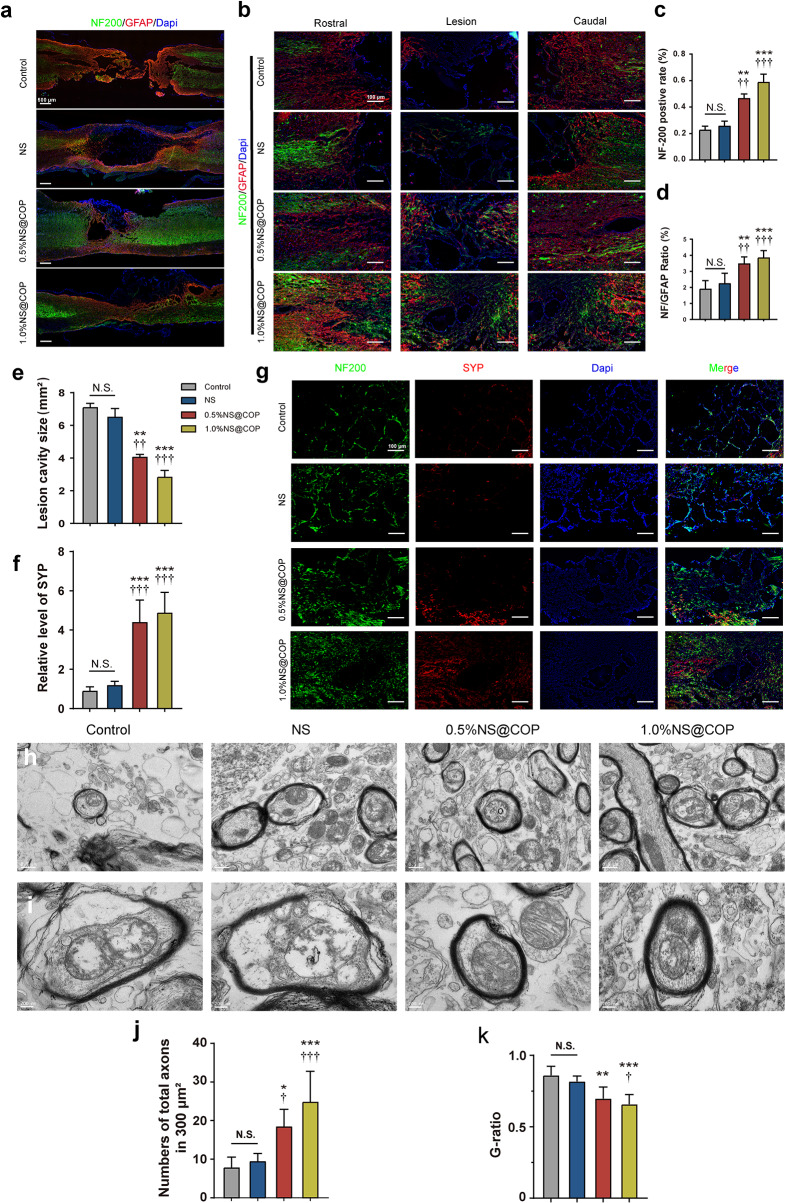
NS@COP prevents cavity formation and enhances neuronal axon regeneration and synaptogenesis. **a**) Representative images of immunofluorescence staining of the full views of the injured spinal cord segments. Spinal cord lesion sites were visualized using NF200 and GFAP staining (*n* = 5). Scale Bar = 500 μm. **b**) Representative images of immunofluorescence staining of spinal cord adjacent to the lesion and in the lesion sites (*n* = 5). Scale Bar = 100 μm. **c-d**) Quantitative analysis of the relative fluorescent intensity of NF200 and NF200/GFAP ratio in the lesion sites. **e**) Size of lesion cavity measured from (a). **f**) Quantitative analysis of the relative fluorescent intensity of SYP in the lesion sites. **g**) Representative images of immunofluorescence staining of NF200 and SYP of spinal cord tissue in control, NS, 0.5%NS@COP, and 1.0%NS@COP groups at 8 weeks post surgery (*n* = 5). Scale Bar = 500 μm. **h**) Ultrastructural images of the transverse sections at the lesion site of the SCI at different groups(*n* = 5).Scare Bar = 0.5 μm. **i**) Representative images of electron microscopy images of the cross sections at the lesion site of the SCI at different groups(*n* = 5).Scare Bar = 200 nm. **j**) Quantitative analysis of the number of axons from TEM images. **k**) Quantitative analysis of the area-based G-ratio from TEM images. Data are presented as mean ± SD. (*) denotes *p* < 0.05, (**) denotes *p* < 0.01, (***) denotes *p* < 0.001, vs. control group: (†) denotes *p* < 0.05, (††) denotes *p* < 0.01, (†††) denotes *p* < 0.001, vs. NS group

To investigate whether NS@COP implantation promotes axon regeneration to aid locomotor function recovery following SCI in vivo, we assessed axon regeneration at the lesion site using protein expression and localization. At 8 weeks post-treatment, severe lesion cavities and a discontinuous spinal cord were observed. However, the 1%NS@COP-treated group showed a significantly reduced lesion cavity compared to the control group (Fig. 9, a and e). We labeled neuronal axons and astrocytes using neurofilament (NF-200) stained in green and glial fibrillary acidic protein (GFAP) stained in red, respectively. NF-200 positive neurofilaments were clearly distributed in the lesion site of the SCI rats treated with 1%NS@COP (Fig. 9b), suggesting enhanced axon growth. Semi-quantitative analysis of the fluorescence intensity of NF-200 in the 1% NS@COP group was significantly higher than in the NS and control groups (Fig. 9c), further supporting this observation. Furthermore, the density and distribution of NF-200 across astrocytes have been proven to be negatively correlated with that of GFAP, suggesting the extent of nerve regeneration [53]. The incorporation of 1%NS@COP induced longer and larger amounts of nerve fibers with mitigated GFAP + astrocyte accumulation (Fig. 9d). The formation of a local neuronal circuit is considered to indicate the recovery of neurobehavioral function. Synaptophysin (SYP), a presynaptic marker that labels connections with nerve fibers, was detected in higher amounts in the lesion region after treatment with 1%NS@COP, indicating that more connections were produced to bridge between different neurons (Fig. 9, f and g).

The ultra-thin sections from the lesion site were harvest in all group, and examined by TEM at 8 weeks post-surgery. In the control group, there were infrequent regenerated axons and myelin sheaths. Quantitative analysis showed that the density of myelinated nerve fibers in the 1%NS@COP group was significantly higher than the SCI group (Fig. 9, h and j).To further assess the extent of re-myelination, the area-based G-ratio (axon area/the whole myelinated axon area) was calculated by ultrastructural analysis using TEM. In the NS@COP groups, the area-based G-ratio (0.66 ± 0.07) was significantly lower than the NS groups (0.82 ± 0.04) and SCI (0.86 ± 0.06) groups, indicating a relatively mature myelin regeneration (Fig. 9, i and k). Additionally, the G-ratio performed that regenerated nerve fibers of the 1%NS@COP groups showed greater myelinated either in small or larger nerve fibers, compared to the NS and SCI groups, reflecting a persistent pro-myelinating ability of the living nerve fibers. We also observed the myelination of axons by IF staining longitudinal sections in the lesion site using myelin basis protein (MBP, indicates myelin sheath) and NF (indicates nerve fiber), respectively (Fig. S7). In the NS@COP group, the images of the lesion sections exhibited the abundant regenerated nerve fibers sheathed with densely and organized myelin. These results are consistent with hindlimb locomotor function outcomes and suggest that 1%NS@COP contributed to functional recovery through its inhibitory effect on glial scar formation and enhancement of nerve regrowth and re-myelination across the lesion area. Furthermore, the results suggest that NS@COP implantation may have the potential to promote axon regeneration and ultimately improve the recovery of locomotor functions after SCI.

## Discussion

Immune microenvironment impairment, energy-supporting deficit, and excessive glial scar formation significantly contribute to the limitation of spinal cord axon regeneration capacity after SCI [19, 46]. Following the initial SCI, a substantial influx of pro-inflammatory macrophages is recruited to the site of trauma, secreting inflammatory cytokines, such as interleukin 1β (IL-1β) and tumor necrosis factor-α (TNF-α). This creates a pro-inflammatory microenvironment, culminating in secondary injury of neural cells, including pyroptosis and apoptosis [54, 55]. Consequently, macrophages emerge as pivotal modulators in maintaining tissue homeostasis and facilitating repair by transforming their phenotype from pro-inflammatory to anti-inflammatory/pro-healing [56]. Numerous previous studies have demonstrated that CGRP, a neuropeptide widely expressed in the dorsal horn of spinal cord, possesses immunotolerant and immunomodulatory properties, which can promote macrophages to transfer to M2 phenotype, an anti-inflammatory phenotype [40, 56–59]. Additionally, CGRP has been shown to protect the CNS against the deleterious effects of inflammation, blood-brain barrier injury, brain oedema, and cognitive decline that occur from cerebral ischemic injury [60–62]. These findings underscore the role of CGRP in promoting CNS protection. Therefore, our next inquiry revolves around whether CGRP can regulate macrophages at the lesion site following SCI.

In our in vitro experiment, we introduced H_2_O_2_ to the medium to mimic the microenvironment of oxidative stress and pre-incubated RAW264.7 cells. Interestingly, the macrophage did not effectively respond to the CGRP immune regulation under excessive oxidative stress environment. In this study, we found that the RAMP1, a vital component of the CGRP receptor, was significantly downregulated in RAW264.7 cells under excessive ROS microenvironment (Fig. 3e and f). More importantly, the synergistic regulation of CGRP was reestablished with RAMP1 up-expression by the ROS scavenging of NS@COP through downstream AKT signaling pathway. Taken together, our study indicated that NS@COP could simultaneously increase RAMP1 expression in macrophages, enabling efficient CGRP/RAMP1/AKT signaling flow to restore relationship between CGRP-mediated M2 polarization (Fig. 3g and Fig. S1-2). Based on these in vitro results, we hypothesized that the CGRP positive nerve/macrophage axis plays a role in the recovery process of SCI. Subsequently, we conducted in vivo experiments and found that transplantation of NS@COP could restore the neuro-inflammatory cross-talk and balance the inflammatory microenvironment after SCI. The NS@COP modulated immune response to neuro-regulation, promoting the polarization of macrophage to M2 phenotype, an anti-inflammatory phenotype, reducing the secretion of pro-inflammatory cytokines, and inhibiting the excessive activation of astrocyte scar, consistent with our results of in vitro studies (Fig. 7 and Fig. S5).

In addition, many studies have reported that excessive ROS could induce not only mitochondrial depolarization and fragmentation but also mitochondrial outer membrane potential dysfunction, leading to necrosis and apoptotic cell death [63, 64]. The membrane potential and ultrastructural changes in mitochondria closely reflected functional changes in cells [65–67]. For successful regeneration, injured axons require energy support in the form of ATP, most of which is supplied by mitochondria in neurons.

All these experimental results collectively demonstrated that NS@COP scaffold exhibited the property of restoring the communication between sensory nerve and macrophage to promote the transition of pro-inflammatory macrophage into an anti-inflammatory pro-resolution M2 phenotype owing to its superior ability to scavenge ROS, and restore neuronal mitochondrial function after SCI, thereby accelerating neural axon regeneration and improving long-term recovery of motor function in vivo. Finally, the pathological sections of major NS@COP organs were performed to preliminary evaluated the in vivo toxicity of NS@COP. As illustrated in Fig. S7, compared with the control group, no discernible histopathological changes, including necrosis, fibrosis and hydroncus, were observed in the pathological sections of heart, liver, lung, spleen and kidney in the NS@COP treatment groups. It is expected that supplemented nanomaterials could provide satisfactory biological properties without significant toxicity in vivo.

## Conclusion

In this study, we constructed an intelligent ROS-responsive nanomaterial for COPs delivery. In both in vitro and in vivo, NS@COP effectively reconstructed the neuroimmune cross-talk to regulate macrophage fate determination toward an M2 anti-inflammatory phenotype and maintained the mitochondrial energy supplying, leading to significantly enhanced neuroprotection and axonal regeneration. The locomotor function of hindlimbs of SCI rat was remarkedly improved following implantation of the scaffold. Our murine studies are limited to motor function settings and lack information regarding other organ function settings, which will be investigated in future studies. However, these future experimental opportunities for continued material optimization highlight the potential of our strategy not only for SCI but also for various other clinical indications that suffer from similar limitations. Last, we hope that our work will contribute to the development of new and effective therapies for patients suffering from spinal cord injuries.

### Electronic supplementary material

Below is the link to the electronic supplementary material.

Supplementary Material 1

## Data Availability

No datasets were generated or analysed during the current study.
