# Supplementary material for: Neuroimmune modulating and energy supporting nanozyme-mimic scaffold synergistically promotes axon regeneration after spinal cord injury
Source: J Nanobiotechnology. 2024 Jul 5;22:399. doi: 10.1186/s12951-024-02594-2 (PMC11225227; doi:10.1186/s12951-024-02594-2)
Supplement: Supplementary file 1 — Supplementary Material 1 [file 12951_2024_2594_MOESM1_ESM.docx]

Table S1 The primer sequences for each primer used in the RT-PCR

| **Primers** | **Forward** | **Reverse** |
| --- | --- | --- |
| IL-1β | TGACCTGTTCTTTGAGGCTGAC | CATCATCCCACGAGTCACAGAG |
| TNF-α | CCAGGTTCTCTTCAAGGGACAA | GGTATGAAATGGCAAATCGGCT |
| IL-4 | TACCAGGAGCCATATCCACGGATG | TGTGGTGTTCTTCGTTGCTGTGAG |
| IL-10  SOD | GAATAGGCCGGTCCAATCAGA  ATGACTTCTTCGTCCCATCCT | CAGCCATTCGTCGGACACATT  CCAACTCCCACATCACCCTTT |
| CAT | AGCTACTGAATCAGCATCCCT | CGCCACTAGCATGTAGAACAAA |
| GAPDH | GGCACAGTCAAGGCTGAGAATG | ATGGTGGTGAAGACGCCAGTA |


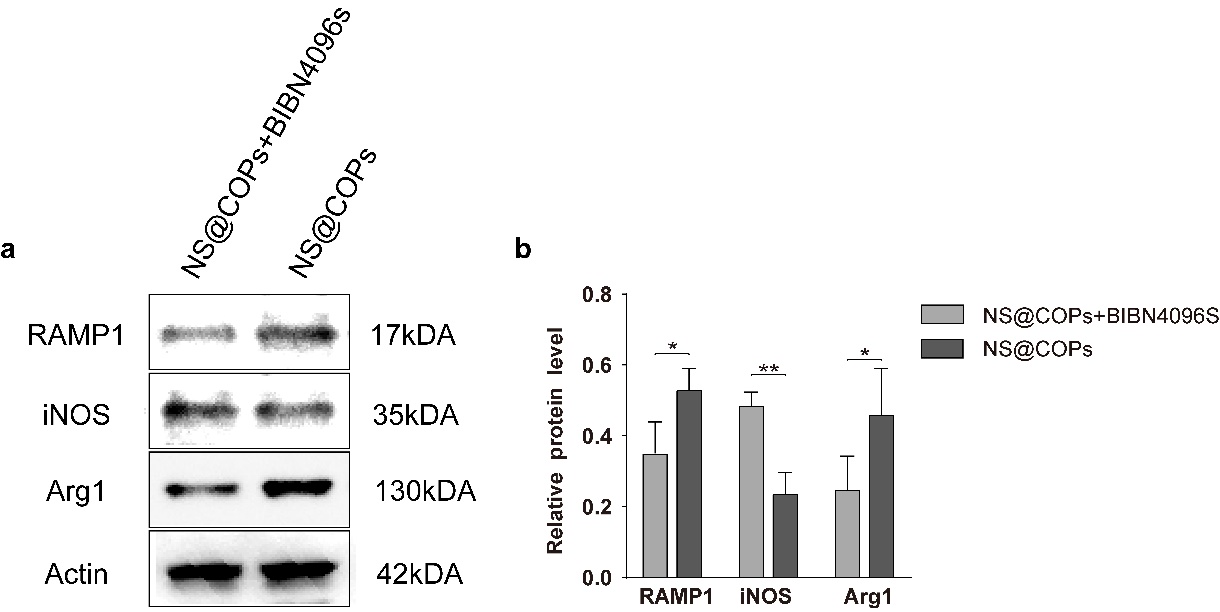


Supplementary Fig. 1 NS@COPs increased RAMP1 level in macrophage. (a) Western blot showing the expression of RAMP1, iNOS, and Arg1 in RAW.264.7 cells treated with or without BIBN4096s after irritated by H_2_O_2_ for 4 hours. (b) Quantitative analysis of relative intensity of RAMP1, iNOS, and Arg1 (n =3). (*) denotes p <0.05, (**) denotes p <0.01.


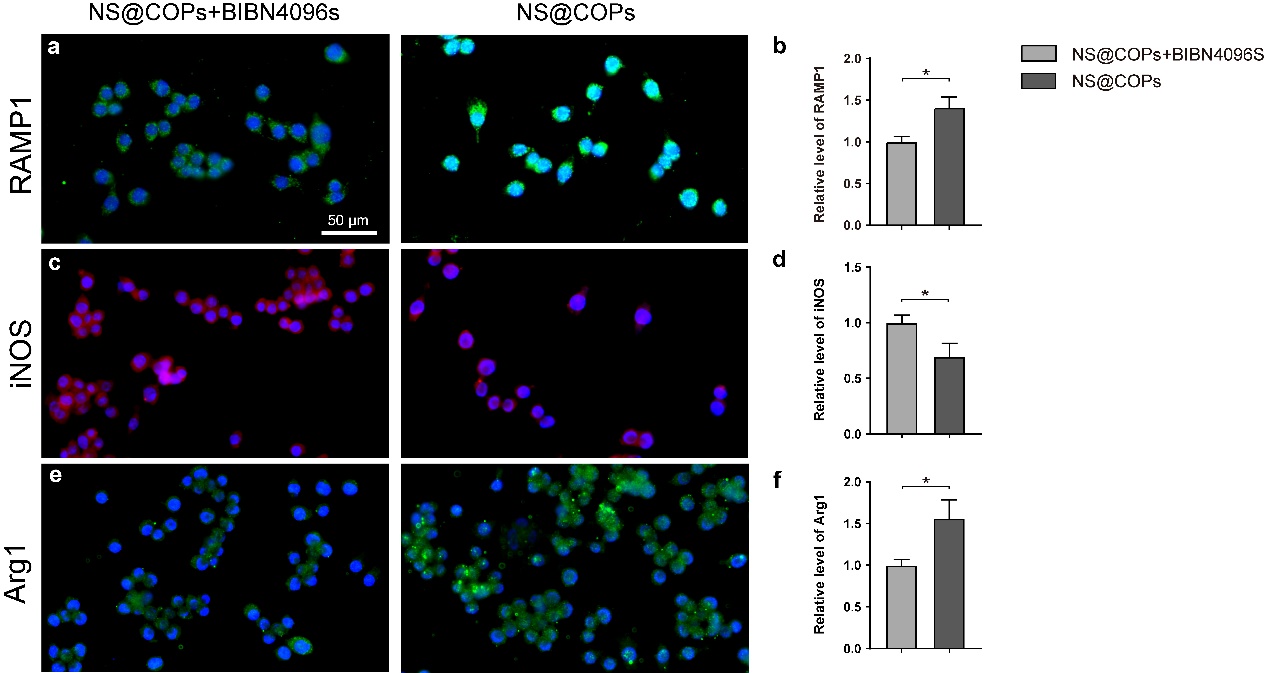


Supplementary Fig. 2 BIBN4096s partially inhibited the protective effect of NS@COP on RAW264.7. a, c, and e) Representative images of immunofluorescence staining of RAMP1, iNOS, and Arg1 in RAW264.7 cells. Scale Bar = 50 μm. b, d, and f) Quantitative analysis of the relative fluorescent level of (a), (b), and (e) (n=3). Data are presented as mean ± SD. (*) denotes p < 0.05, (**) denotes p < 0.01, (***) denotes p < 0.001.


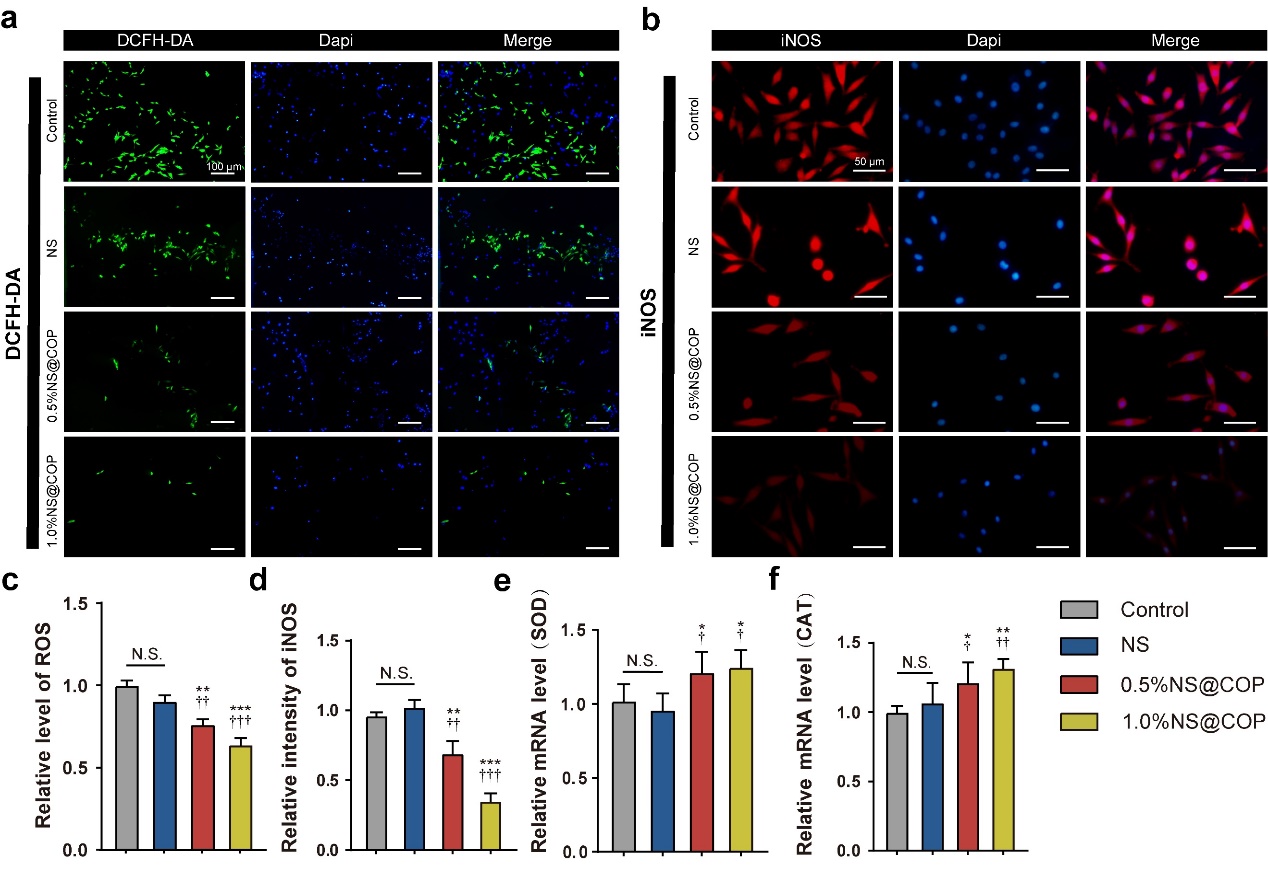


Supplementary Fig. 3 NS@COP alleviates H_2_O_2_-induced oxidative stress of cells *in vitro*. a, b) Representative staining images of intracellular ROS was detected by DCFH-DA probe and iNOS antibody, respectively. c, d) Quantitative analysis of the relative fluorescent level of (a)-(b) (n=3). e, f) RT-PCR analysis of the intracellular SOD and CAT mRNA expression levels after co-culturing with NS, 0.5%NS@COP, and 1%NS@COP scaffold, respectively. Data are presented as mean ± SD. (*) denotes p < 0.05, (**) denotes p < 0.01, (***) denotes p < 0.001, vs control group: (†) denotes p < 0.05, (††) denotes p < 0.01, (†††) denotes p < 0.001, vs NS group.


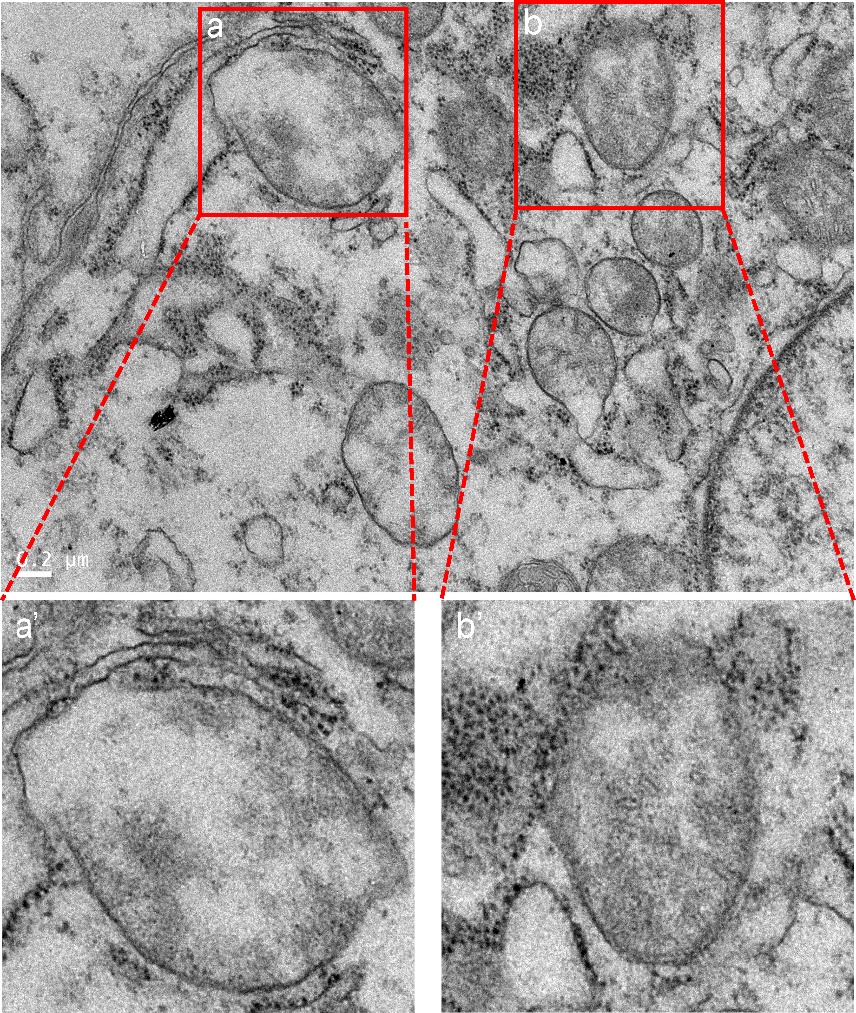


Supplementary Fig. 4 COPs accumulate at the mitochondrial outer mitochondria and the inner leaflet of the plasma membrane in cultured cells. Scale Bar = 0.2 μm


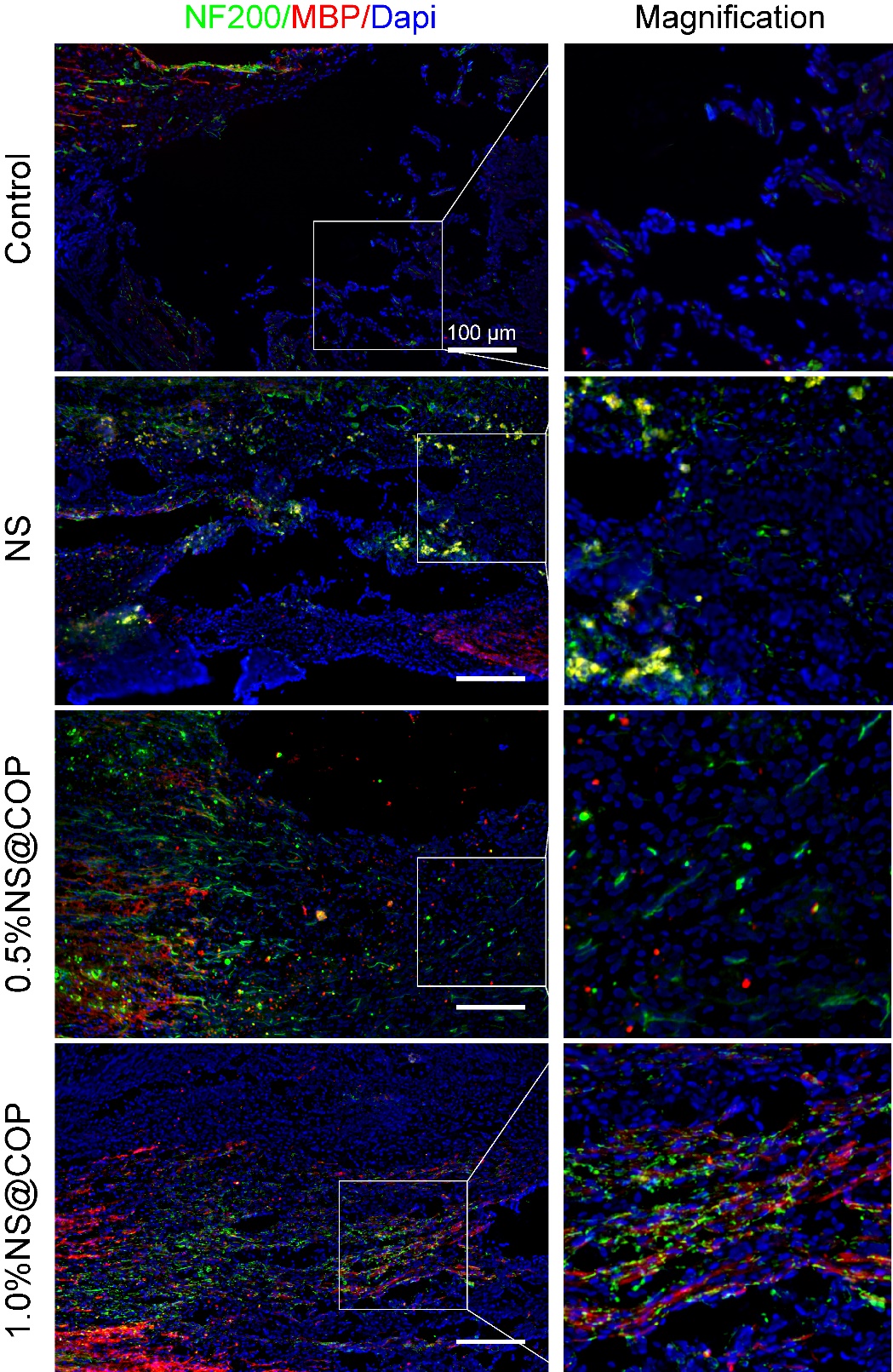


Supplementary Fig.5 Representative images of immunofluorescence co-staining of NF200 (green) and MBP (red) of spinal cord tissue in control, NS, [0.5%NS@COP](mailto:0.5%25NS@COP), and 1.0%NS@COP groups at 8 weeks post surgey. Scale Bar = 100 μm.


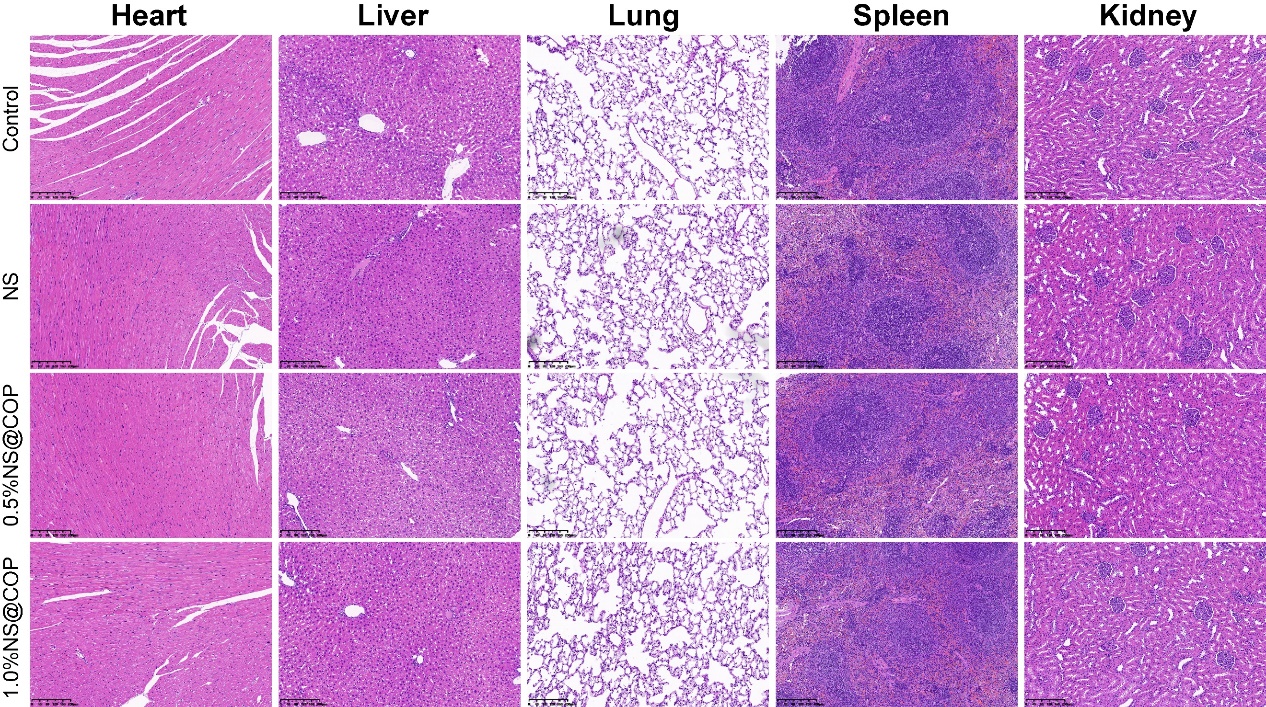


Supplementary Fig.6 Verification of biological safety of NS and NS@COP *in vivo*. Morphology of the major function organ (heart, liver, lung, spleen, and kidney. Scale Bar = 200 μm.


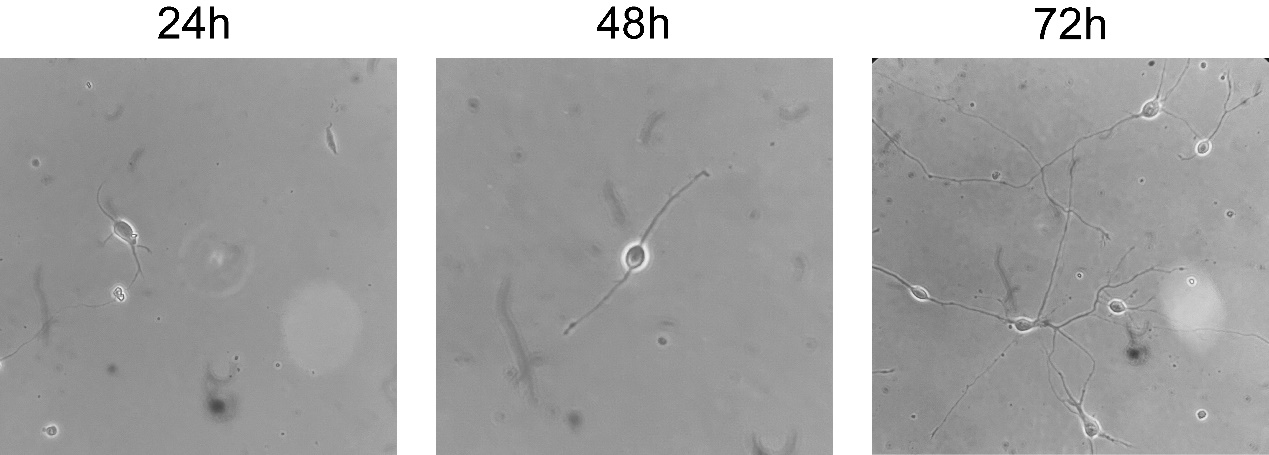


Supplementary Fig. 7 Representative images of the neurons under bright-field.
